# A Short Receptor Downregulates JAK/STAT Signalling to Control the *Drosophila* Cellular Immune Response

**DOI:** 10.1371/journal.pbio.1000441

**Published:** 2010-08-03

**Authors:** Rami Makki, Marie Meister, Delphine Pennetier, Jean-Michel Ubeda, Anne Braun, Virginie Daburon, Joanna Krzemień, Henri-Marc Bourbon, Rui Zhou, Alain Vincent, Michèle Crozatier

**Affiliations:** 1 Université Toulouse 3, Toulouse, France; 2 Centre de Biologie du Développement UMR5547 CNRS Toulouse, France; 3 Institut de Biologie Moléculaire et Cellulaire, UPR9022 CNRS, Strasbourg, France; 4 Department of Genetics at Harvard Medical School, Boston, Massachusetts, United States of America; Stanford University, United States of America

## Abstract

The posterior signalling centre (PSC), a small group of specialised cells, controls hemocyte (blood cell) homeostasis in the *Drosophila* larval hematopoietic organ, the lymph gland. This role of the PSC is very reminiscent of the “niche,” the micro-environment of hematopoietic stem cells in vertebrates. We have recently shown that the PSC acts in a non–cell-autonomous manner to maintain janus tyrosine kinase/signal transducers and activators of transcription (JAK/STAT) signalling in hematopoietic progenitors (prohemocytes), thereby preserving the multipotent character necessary for their differentiation into lamellocytes, a cryptic and dedicated immune cell type required to fight specific immune threats such as wasp parasitism. In this report, on the basis of a knock out generated by homologous recombination, we show that a short type I cytokine-related receptor CG14225/Latran is required for switching off JAK/STAT signalling in prohemocytes. This is a prerequisite to massive differentiation of lamellocytes upon wasp parasitisation. In vivo and cell culture assays indicate that Latran forms heteromers with Domeless, the *Drosophila* type I cytokine signalling receptor related to mammalian GP130, and antagonises Domeless activity in a dose-dependent manner. Our analysis further shows that a primary immune response to wasp parasitism is a strong decrease in cytokine mRNA levels in the lymph gland, followed by an increase in the *latran/domeless* ratio. We propose that this sequence of events culminates in the complete inhibition of residual JAK/STAT signalling by Latran. JAK/STAT activity has been associated with several human diseases including leukaemia while knock-out studies in mice point to a central role of this pathway in hematopoiesis and regulation of immune functions. The specific function of *Drosophila* Latran is, to our knowledge, the first in vivo example of a role for a nonsignalling receptor in controlling a dedicated immune response, and thus raises the question of whether short, nonsignalling receptors also control specific aspects of vertebrate cellular immunity.

## Abstract 2

Regulation of JAK/STAT signalling by a short, nonsignalling receptor in *Drosophila* modulates response to specific immune challenges such as parasitoid infestations.

## Author Summary

A specific microenvironment termed the “niche” supports long term maintenance of hematopoietic stem cells in vertebrates. A small group of specialised cells called the posterior signalling center (PSC) controls hemocyte (blood cell) homeostasis in the *Drosophila* larval hematopoietic tissue and thus fulfills a similar function to the vertebrate niche. The PSC acts at a distance to maintain JAK/STAT signalling in hematopoietic progenitors (prohemocytes), thereby ensuring their multipotent character. We report here that a short cytokine receptor encoded by *CG14225/latran* is required to extinguish JAK/STAT signalling in prohemocytes and thereby ensures their mass differentiation into lamellocytes, an immune cell type required to fight specific threats such as wasp parasitism. Domeless, a related receptor in *Drosophila*, was previously the only known cytokine receptor that signals through the JAK/STAT pathway. We show that Latran lacks the intracellular domains required for signal transduction and acts instead by antagonizing the function of Domeless in a dose-dependent manner. The role of *Drosophila* Latran in the repression of JAK/STAT signalling under specific immune conditions raises the question of whether short, nonsignalling receptors that antagonize full-length receptors could also control specific aspects of vertebrate immunity.

## Introduction

**Figure 1:**
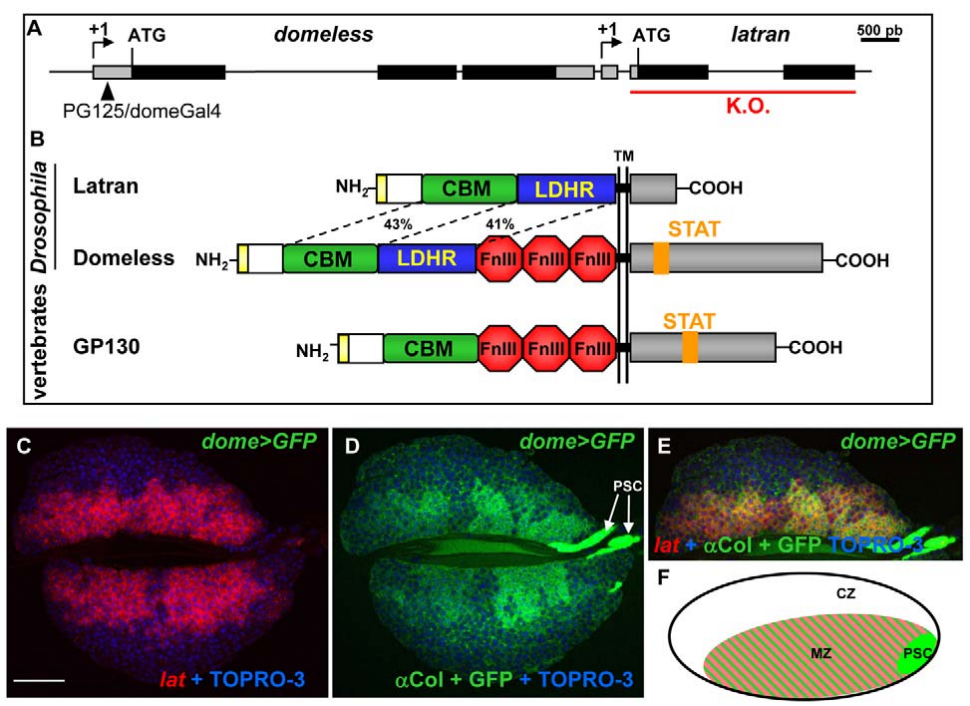
Latran: homology with JAK/STAT receptors and expression in the LG. (A) Schematic of the *D. melanogaster* dome-lat genomic region. The ORFs and untranslated 5′ and 3′ regions are indicated by black and grey boxes, respectively. Positions of the PG125/domeGal4 P-element insertion [67] and the lat genomic fragment that was deleted to generate a null allele are indicated by an arrowhead and a red bar, respectively. (B) Schematic alignment of the Lat, Dome, and human GP130 proteins. The green box corresponds to the CBM and the blue box to a conserved region between Lat and Dome LDHR; the percentage of sequence identity is given. Fibronectin III (Fn III) motifs are indicated in red, the signal peptide in yellow. TM indicates the transmembrane domain. The intracytoplasmic regions are in grey, the position of the STAT binding site in orange. (C–E). Overlapping expression of lat (ISH red) (C), dome>GFP (GFP, light green) and Col (Ab, bright green, arrows) (D), and triple overlap (E) in LGs of a third instar larva. Nuclei are highlighted in blue (TOPRO-3); anterior is to the left. Scale bar, 80 µm. (F) Schematic of an anterior lobe of the LG.

The innate immune response–the synthesis of antimicrobial peptides and mobilisation of dedicated immune cells–confers a broad protection against pathogens to all multicellular organisms. *Drosophila* has become a model for studying the role of hematopoietic (blood) cells and the evolution of cellular immunity (reviews by [1],[2]). Similar to vertebrates, *Drosophila* hematopoiesis occurs in two waves during development [3]. A first population of hemocytes is specified in the embryo and gives rise to plasmatocytes involved in phagocytosis and crystal cells required for melanisation [4]. A second wave of plasmatocyte and crystal cell production occurs at the end of larval development. Larval hematopoiesis can also give rise to a third cell type, the lamellocytes, which are devoted to the encapsulation of foreign bodies too large to be phagocytosed. Lamellocytes only differentiate in response to specific immune challenges such as parasitisation by wasps, a common threat for higher order insects [1],[2],[5],[6]. Larval hematopoiesis takes place in a specialised organ, the lymph gland (LG), which forms during embryogenesis and grows during larval development. In third instar larvae, the LG is composed of several lobes with the anterior-most lobes organised into a medullary zone (MZ) containing the hematopoietic progenitors, a cortical zone (CZ) containing differentiating hemocytes, and a so-called posterior signalling centre (PSC) (Figure 1F) [7]. These three zones can be identified by the expression of different markers. Differentiating crystal cells and plasmatocytes in the CZ express prophenoloxidase (proPO) and the P1 Nimrod receptor [8], respectively. Hematopoietic progenitors can be distinguished by their expression of *domeless* (*dome*), which encodes the *Drosophila* receptor of the janus tyrosine kinase/signal transducers and activators of transcription (JAK/STAT) pathway and *tep4*, which encodes a thioester protein [7],[9]. PSC cells express the transcription factors Antennapedia (Antp) and Collier/Knot (Col), the *Drosophila* Early B-cell Factor (EBF) ortholog [10], and the morphogen Hedgehog (Hh) [7],[11]–[13]. The PSC controls the balance between multipotent prohemocytes present in the MZ and differentiating hemocytes [9],[12]. It acts in a non–cell-autonomous manner, perhaps via Hh signalling, to maintain JAK/STAT signalling in prohemocytes, thus preserving the multipotent character necessary for these cells to adopt a lamellocyte fate upon parasitisation. This key role of the PSC revealed that, in *Drosophila*, larval hemocyte homeostasis is dependent upon interactions between hematopoietic progenitors and their micro-environment, a role reminiscent of the vertebrate hematopoietic niche [9],[12],[14].

JAKs and STATs mediate intracellular signalling in response to secreted type I cytokines [15]. In mammals, large families of cytokines and single-pass transmembrane receptors, named type I cytokine receptors, which signal as either homodimers or heteromers, have been identified (for review [16]–[19]). JAK kinases are anchored to the intracellular part of signalling receptors. Binding of the cytokine induces conformational changes in the latter that bring two JAKs in close proximity. This allows JAK trans-phosphorylation and phosphorylation of the receptor, thereby creating a docking site for STAT transcription factors. STATs become in turn phosphorylated, leading to their dimerisation and translocation into the nucleus where they function as transcriptional regulators. Recent finding in *Drosophila* also point to a noncanonical mode of JAK/STAT signalling, which could directly control heterochromatin stability (for review [20]). Altered JAK/STAT activity has been associated with several human diseases including leukaemia, myocardial hypertrophy, and asthma, while knock-out studies in mice point to a central role in hematopoiesis and regulation of immune functions [21],[22]. In contrast to mammals, only one receptor, Domeless (Dome), one JAK (Hopscotch, Hop), one STAT (Stat92E or Marelle) and three cytokines, Unpaired (Upd), Upd2, and Upd3 have been functionally characterised in *Drosophila* (for review [23]). Sequencing of the *D. melanogaster* genome revealed, however, the existence of a *dome*-cognate gene (*CG14225*, renamed here *latran* [*lat*]) (Figure 1) [24],[25].

We report here that *lat* acts as a negative regulator of the JAK/STAT pathway during larval hematopoiesis. *lat* is required for turning off JAK/STAT signalling in hematopoietic progenitors following wasp parasitisation, thereby allowing the massive differentiation of lamellocytes. In vivo and in vitro assays indicate that Latran (Lat) forms heteromers with Dome and antagonises Dome function in a dose-dependent manner. Our studies thus revealed a novel mode of regulation of JAK/STAT signalling, based on differential and tissue-specific expression of signalling and antagonist cognate receptors. The tight tissue-specific regulation of JAK/STAT signalling by *latran* is crucial for *Drosophila* to be able to mount a dedicated cellular immune defense. A negative regulation of JAK/STAT signalling by a nonsignalling receptor chain has, so far, only been reported in primary and cultured mammalian cells, for short versions of class I cytokine receptors [26],[27]. However, the in vivo function of these short membrane receptors and how their expression is regulated and linked to tissue homeostasis remain to be established. The specific role of *Drosophila lat* in controlling a dedicated cellular immune response raises the possibility that nonsignalling receptors could control specific aspects of vertebrate immunity, prefiguring a new field of investigations on this pathway.

## Results

### *CG14225/latran* Encodes a JAK/STAT Receptor-Like Protein

Vertebrate class I (one) cytokines bind to receptors composed of various single-pass transmembrane protein chains that form homo- and heteromeric complexes. Dome is the only class I cytokine receptor that has, so far, been characterised in *Drosophila* [28]–[31]. Existence of a *D. melanogaster* gene, *CG14225/lat*, coding for a protein structurally related to Dome was noticed several years ago [24]. *dome* and *lat* are adjacent to each other on the X chromosome and transcribed in the same orientation, suggesting that they originated from a gene duplication event (Figure 1A). The key role of JAK/STAT signalling in regulating larval hemocyte homeostasis [9],[32] led us to ask whether *lat* was involved in this regulation. We first experimentally defined the 5′ end of *lat* transcripts by RACE-PCR, using total RNA from LGs. We positioned the *lat* methionine initiation codon and established that 153 bp separate the 3′ end of *dome* mRNAs from the *lat* transcription start site (Figures 1 and S1A). Dome and Lat show strong similarity in their extracellular domains, which include, from N- to C-terminal, a signal peptide, a cytokine binding motif (CBM) related to that of vertebrate receptors, and an approximately 200 amino acid region not found in vertebrate receptors (Figures 1B and S2). We designate this region, whose function remains unknown, as LDHR for Lat-Dome Homology Region. Similar to the human class I cytokine receptor GP130, Dome contains three tandemly arranged fibronectin type III (Fn III) motifs. None Fn III motif are found in Lat. The intracellular region of Lat is shorter than that of Dome and shows no consensus STAT binding site (motif YXXQ, [25]) suggesting that *lat* encodes a nonsignalling form of cytokine receptor. A search for *dome/lat*-related genes in available genomes of other *Drosophila* species indicated that *lat* and *dome* are arranged in tandem, with an intergenic region varying from 150 bp (*D. melanogaster*) to 2.5 kb (*D. virilis*) (Figure S3). A higher degree of sequence conservation between orthologous compared to paralogous genes was observed (Figure S3), suggesting that *lat* sequences diverge at a much higher rate than those of *dome*.

### *lat* Is Specifically Expressed in Hematopoietic Progenitors in the *Drosophila* LG

In situ hybridisations show that unlike *dome*, *lat* is not expressed in embryos, a result confirmed by reverse transcriptase PCR (RT-PCR) (unpublished data). Thus, despite genomic proximity, the control of *lat* transcription is different from that of *dome*. In larvae, *lat* transcription was detected only in pericardial and LG cells. In situ hybridisation onto LGs expressing a membrane targeted green fluorescent protein (GFP) either in the MZ (dome-Gal4/UAS-mcd8 GFP (dome>GFP)) or the PSC (pcol85/UAS-mcd8GFP (pcol>GFP)) indicated that *lat* is only expressed in the MZ (Figure 1C–1F). Coexpression of *dome* and *lat* in MZ cells, where JAK/STAT signalling is critically required to maintain a pool of hematopoietic progenitors [9], raised the question of the role of *lat* in controlling the activation of the JAK/STAT pathway in prohemocytes.

### *lat* Mutants Are Fully Viable but Unable to Mount a Cellular Immune Response against Wasp Parasitisation

**Figure 2:**
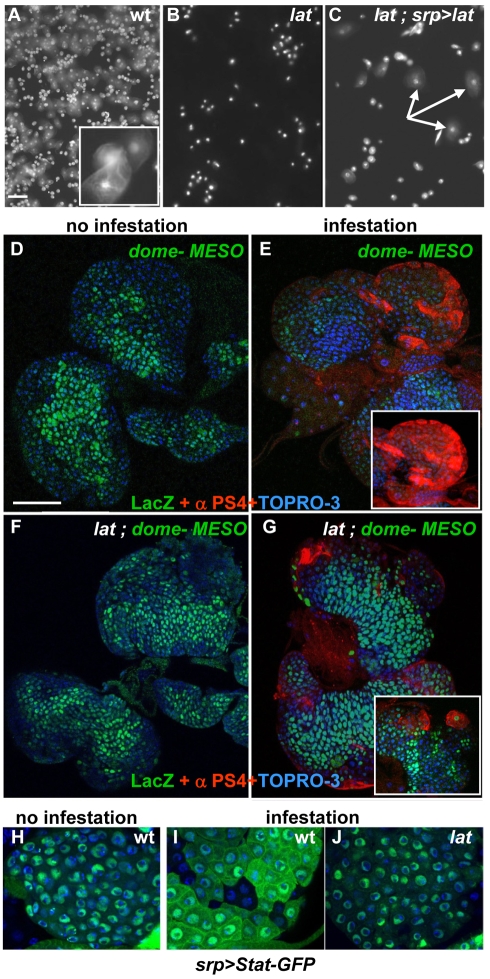
*lat* mutant LGs do not properly respond to wasp parasitisation. (A–C) DAPI staining of circulating hemocytes from (A) wt, (B) *lat* mutant, and (C) *lat* mutant larvae expressing *lat* in the LG (srp>*lat*). 48 h after parasitisation, lamellocytes are found in large numbers in the hemolymph of wt larvae (A, insert) but lacking in *lat* mutants. This lack-of-lamellocyte phenotype is rescued by expressing *lat* in the LG (C, arrows). (D–I) Prohemocytes, marked by dome-MESO expression (green) are not maintained in wt LGs following wasp parasitisation (D and E); this is paralleled by massive lamellocyte differentiation (integrin α chain [α-PS4], red) (E, insert). In *lat* mutant larvae, dome-MESO remains expressed after parasitisation (F, G); few lamellocytes differentiate (G, insert). (H–J) Stat-GFP (green) subcellular localisation in wt (H and I) and *lat* mutant LG (J), either without (H) or 4–6 h after parasitism (I and J). *srp*-Gal4 was used to drive UAS-Stat-GFP in the LG (srp>Stat-GFP). Stat-GFP localisation is nuclear in nonparasitised wt and parasitised *lat* LG, whereas it is both cytoplasmic and nuclear in parasitised wt LG. The scattered distribution of GFP-labelled cells is due to nonuniform activation of the *srp*-Gal4 driver in the LG [11]. Nuclei (TOPRO-3) are in blue. Scale bars, 50 µm (A); 80 µm (D).

To determine if *lat* plays a role in larval hematopoiesis, we generated a *lat* null allele by homologous recombination [33]. Several independent recombination events were obtained and homozygous mutant lines were established (Figure S1). Homozygous and trans-heterozygous combinations of these lines produced fertile adults with no obvious morphological defects, indicating that *lat* is not essential for either viability or germ-line development. In particular, no phenotypic defect was observed in the eye, where the JAK/SAT pathway plays an important role in growth and patterning [34],[35]. We then looked at the morphology of the LG in *lat* mutant larvae, using specific markers for the MZ (*tep4*), the PSC (*col*), or for differentiated hemocytes: crystal cells (*doxA3*) and plasmatocytes (P1). No obvious difference could be found between wild type (wt) and *lat* mutant larvae, suggesting that *lat* is neither required for the ontogeny of the LG, nor for the differentiation of plasmatocytes and crystal cells (Figure S4). The third type of *Drosophila* hemocytes, the lamellocytes (identified by the integrin α chain [α-PS4 marker]), massively differentiate at the expense of the pool of hematopoietic progenitors upon wasp parasitisation; they start to differentiate in the LG before being released into the hemolymph. In wt larvae, the number of circulating lamellocytes reaches its maximum 48 h after wasp egg-laying [5]. In sharp contrast to wt, virtually no circulating lamellocytes are found in the hemolymph of parasitised *lat* mutant larvae (Figure 2A, 2B), either 48 or 72 h after wasp egg-laying. Several days later, adult wasps hatch from parasitised *lat* mutant pupae. *srp*-Gal4 driven *lat* expression in the LG (*srp*>*lat*) completely restored the ability of *lat* mutant larvae to produce lamellocytes following wasp parasitisation (Figure 2C). We therefore conclude that *lat* is required for the massive differentiation of lamellocytes in response to wasp parasitisation.

Lamellocyte production upon parasitisation involves downregulation of JAK/STAT signalling in the MZ, thereby licensing hematopoietic progenitors to differentiate [9]. JAK/STAT activity in the LG can be monitored by the expression of a reporter transgene, *dome-MESO-lacZ* (dome-MESO), where LacZ is under the control of an intronic *dome* regulatory element [9],[36],[37]. Under normal conditions, LacZ expression is observed in the MZ of *lat* mutant as in wt larvae, indicating that the JAK/STAT pathway is active and that *lat* is not required for this activity (Figures 2 and S4). 30 h postinfestation a strong reduction of dome-MESO expression is observed in wt LGs (Figure 2D, 2E) correlating with lamellocyte differentiation (Figure 2E, insert) and premature LG dispersal [5]. In sharp contrast, dome-MESO remains expressed in *lat* mutant LGs and these, unlike wt LG, do not prematurely disperse (Figure 2F, 2G), correlating with the absence of circulating lamellocytes in the hemolymph. This shows that *lat* is required for the downregulation of JAK/STAT activity in hematopoietic progenitors following parasitisation. The observation of few differentiated lamellocytes in *lat* mutant larvae (Figure 2G and insert) indicates, however, that *lat* is not required for the lamellocyte differentiation program *per se*. In cells were the JAK/STAT pathway is activated, Stat has a predominantly nuclear localisation. In order to follow the activity of the pathway after parasitism, we analysed the subcellular localisation of a fluorescent Stat protein, Stat-GFP [38], expressed in the LG (*srp-Gal4;STAT92E-GFP*). In noninfectious conditions, Stat-GFP is mainly found in the nuclei, in either wt (Figure 2H) or *lat* (unpublished data) mutant larvae, consistent with active signalling. 4–6 h after wasp egg-laying, Stat-GFP is found both in the cytoplasm and the nucleus in wt LG, whereas it remains predominantly localised in the nucleus in *lat* mutant LG (Figure 2I, 2J). These data show a decreased activity of JAK/STAT signalling in wt LG, already 4–6 h after wasp parasitisation, whereas no change can be detected in *lat* mutant LG.

### Lat and PSC Activity in the LG: Robustness of Hemocyte Homeostasis

The PSC (niche) is critically required to maintain a balance between JAK/STAT-positive progenitors and JAK/STAT-negative differentiating hemocytes in third instar LG [9]. The function of *lat* in the MZ raised the question of the relative contribution of positive and negative regulation by the PSC and *lat*, respectively, in the maintenance of this balance. Therefore, we examined the proportion of prohemocytes (expressing dome-MESO) and differentiating hemocytes in LGs double mutant for *lat* and *col* (Figure S5). Whereas in *col* mutant LGs, which lack a PSC, the MZ disappears and all prohemocytes differentiate [9], we observed a less severe phenotype in *lat;col* double mutants, namely the loss of an organised MZ with remaining prohemocytes intermingled with differentiated hemocytes (Figure S5A). Intermingling of prohemocytes and differentiated hemocytes was also observed in *lat;col* double mutants following wasp parasitisation with, in this case, some lamellocytes among differentiated hemocytes (Figure S5B). The persistence of prohemocytes in the *lat;col* double mutant LG underlines the crucial role of *lat* in the complete switch from progenitor to differentiated state that is observed either in *col* mutant larvae or following parasitisation.

### Lat Is a Negative Regulator of JAK/STAT Signalling

**Figure 3:**
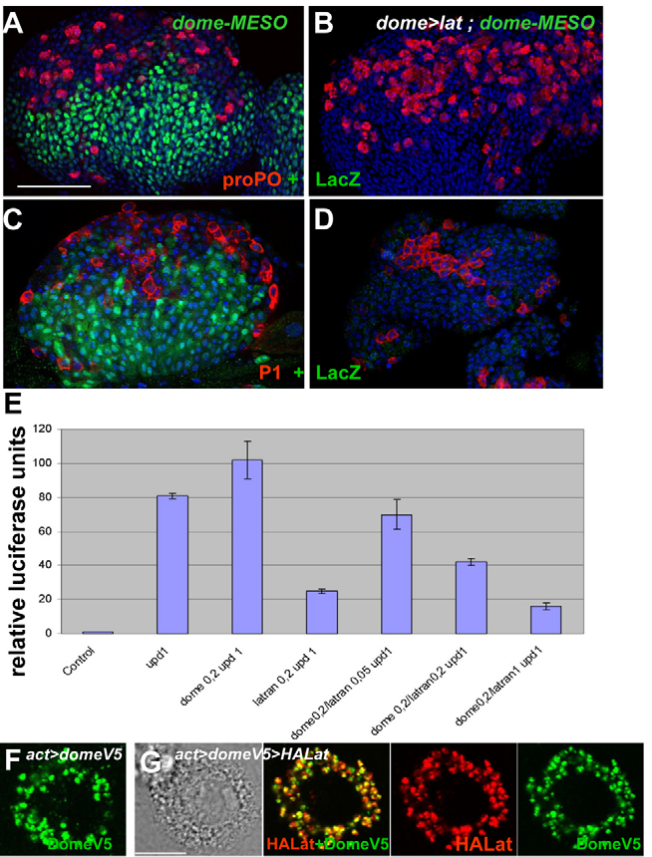
Lat acts as a dominant-negative JAK/STAT receptor. (A–D) dome-Gal4 driven expression of *lat* in the MZ switches off JAK/STAT signalling, as monitored by dome-MESO expression (LacZ, green). proPO (red, A and B) and P1 stainings (red, C and D) indicate differentiating crystal cells and plasmatocytes, respectively. (E) *Drosophila* S2-NP cells were transfected with 10×STAT92E-luciferase, Act-*Renilla*, and Act-*upd*, and various amounts of Act-*dome* and Act-*lat* plasmids (indicated in nanograms). Luciferase assays were performed 4 d later and the reporter activity normalised as the ratio of firefly luciferase/Renilla. The results are from three independent experiments. Vertical bars correspond to SD. (F–G) Immunodetection of Dome-V5 (green) and HA-Lat (red) in *Drosophila* S2-NP cells, in conditions where the JAK/STAT pathway is either on (F) or off (G) (MM and Figure 3E). HA-Lat and Dome-V5 colocalise in trafficking cytoplasmic vesicles in both conditions. (G) Shows a phase-contrast view of a transfected cell. Scale bars, 80 µm (A); 8 µm (G).

The structural similarity between Lat and Dome together with *lat* function suggested that *lat* encodes a novel negative regulator of the JAK/STAT pathway. To test this hypothesis, we overexpressed *lat* in the MZ and followed JAK/STAT activity using dome-MESO expression. *lat* overexpression led to a complete inhibition of JAK/STAT signalling in the MZ while both crystal cells and plasmatocytes were still able to differentiate (Figure 3A–3D). To further investigate the possible mechanism behind this inhibition, we turned to reporter assay developed in cultured *Drosophila* Schneider (S2-NP) cells [29]. S2-NP cells display a basal level of endogenous JAK/STAT activity, as shown by transfection of a STAT reporter gene (10XStat92E-Luciferase reporter). A much stronger activity is observed upon coexpression of either of the cytokines Upd, Upd2 [29],[30],[36], or Upd3 (Figure S6). To assess for *lat* function, we transfected S2-NP cells with 10XStat92E-luciferase, Actin promoter-driven Renilla luciferase, Upd expression vectors (Act-renilla and Act-upd), together with Actin promoter-driven Dome (Act-dome) and/or Lat (Act-lat) expression vectors at various relative concentrations. Since high level of forced Dome expression could act as a dominant-negative [39], we transfected low levels of Act-dome, which modestly increased JAK/STAT signalling (Figure 3E). In contrast, transfection of similar amounts of Act-lat severely decreased signalling (>4-fold), confirming that *lat* acts as a negative regulator of the pathway (Figure 3E) without affecting the level of Dome expression (unpublished data). Lat function is independent of the added cytokine (Figure S6). Intermediate levels of JAK/STAT inhibition were observed for different relative amounts of Act-dome and Act-lat indicating that the ratio between Lat and Dome is critical (Figure 3E). These data both confirmed that *lat* is a negative regulator of JAK/STAT signalling and suggested that the ratio between Dome and Lat is a key factor in controlling JAK/STAT activity.

Binding of Upd to Dome induces endocytosis of receptor-ligand complexes and their trafficking through the endosomal compartments, a trafficking required to activate JAK/STAT signalling [40]. We looked at the intracellular localisation of a tagged Dome (Dome-V5) in cells transfected with both Upd and Act-Dome-V5, where the pathway is active. Dome(-V5) was localised in cytoplasmic vesicles as previously described (Figure 3F) [40]. This localisation was unchanged in cells cotransfected with a tagged Lat (Act-HA-Lat), which results in inactivation of the pathway (Figure 3F, 3G). Both Dome-V5 and HA-Lat were localised in mostly overlapping intracytoplasmic vesicles, indicating that negative regulation of Dome activity by Lat is not linked to a defect in Dome internalisation.

### Lat and Dome Can Form Heterodimers In Vivo

**Figure 4:**
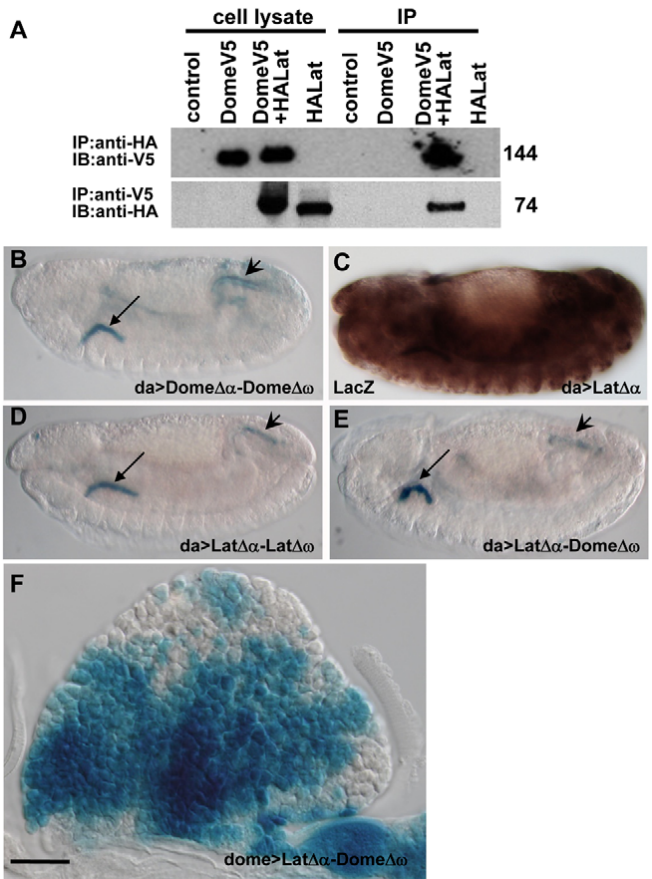
Lat forms heterodimers with Dome in the LG. (A) Immunoprecipitation of the Lat/Dome protein complex. Lysates from either control S2-NP cells or cells transfected with pAc-DomeV5 and pAc-HALat were immunoprecipitated with either anti-V5 or anti-HA antibodies as indicated above each lane and subjected to Western blot analysis. Both conditions indicate the formation of Dome/Lat protein complexes. (B, D–E) Formation of Dome homodimers, Lat homodimers, and Lat/Dome heterodimers in (B) da-Gal4xUAS-DomeΔα/UAS-DomeΔω, (D) da-Gal4; UAS-LatΔα/UAS-LatΔω, and (E) da-Gal4; UAS-LatΔα/UAS-DomeΔω embryos, respectively, as visualised by X-Gal staining (blue). In stage 13 embryos, strong staining is observed in the salivary glands (black arrow) and the hindgut (black arrowhead). (C) da-Gal4; UAS-LatΔα ubiquitous expression of LatΔα detected by LacZ immunostaining. (F) Formation of Dome/Lat heterodimers in the MZ of the LG. Scale bars, 40 µm (F).

Long and short forms of vertebrate class 1 cytokine receptors can form heterodimers/multimers [17],[41]. While Dome was previously shown to form homodimers [42], we tested the possibility that Lat could form heteromers with Dome, by using coimmunoprecipitation (co-IP) assays. S2-NP cells were transfected with equivalent amounts of plasmids encoding tagged Dome(-V5) and Lat(-HA). Cell lysates were prepared 72 h post-transfection and subjected to IP with either anti-V5 or anti-HA antibodies, followed by Western blot analysis with one or the other antibody. Lat and Dome co-IP in both directions indicated that they form heteromers in cell culture (Figure 4A). We then tested whether Lat and Dome form heteromers in vivo, using the βblue-βblau β-galactosidase complementation technique developed to detect protein-protein interactions in vivo [43]–[45] and already applied to show that Dome forms homodimers [42]. Briefly, this technique uses two β-Gal mutant forms (Δα and Δω) that are separately inactive but can complement each other if brought into proximity by fusing them to proteins that physically interact. Like for Dome [42], Δα and Δω β-galactosidases were fused to the Lat C terminus. We used the *da*-Gal4 driver to coexpress different combinations of Lat and Dome fusion proteins in embryos (Figure 4B–4E), because it leads to strong ubiquitous embryonic expression of the proteins (Figure 4C and unpublished data) [42]. Contrasting with this ubiquitous expression, X-gal staining was only detected in the salivary glands and hindgut when Dome Δα and Dome Δω were coexpressed as previously reported (Figure 4B) [42]. A similar staining pattern was observed upon coexpression of LatΔα/LatΔω LatΔα/DomeΔω or DomeΔα/LatΔω (Figure 4D, 4E, and not shown), while no staining could be detected when only one fusion protein was expressed. These results indicate that Lat is able to form homodimers and heterodimers with Dome in vivo. We then tested β-gal complementation in the MZ using the *dom*e-Gal4 driver because *da*-Gal4 is not expressed in the LG (unpublished data). Staining was observed upon coexpression of LatΔα and DomeΔω (Figure 4F) or DomeΔα and LatΔω (unpublished data), while no staining could be observed upon expression of either fusion protein alone. These complementation assays show that Dome and Lat form heterodimers in the LG. Unfortunately we could not determine whether Dome or Lat can also form homodimers in the LG since *dome*-Gal4 driven expression of two copies of either Dome or Lat leads to early larval lethality. We observed, however, that *dome*-Gal4-driven expression of one copy of either Lat, LatΔα or LatΔω resulted in an “outstretched wing” phenotype in adult flies, a phenotype previously described for *upd* mutant flies, hence the name *outstretched (os/upd)* given to these mutants [46],[47]. This observation indicates that both the native and Lat fusion proteins are able to downregulate the JAK/STAT pathway. *eyeless*-Gal4 driven expression of *lat* in eye discs led to smaller eyes, another phenotype reminiscent of *upd* mutants (Figure S7) [39], confirming that *lat* is able to downregulate JAK/STAT signalling in tissues other than the LG when ectopically expressed. Taken together, cell culture and in vivo data indicate that Lat forms inactive heteromers with Dome and is a novel negative regulator of the JAK/STAT pathway.

### Wasp Infestation Results in an Increased Lat/Dome Ratio

**Figure 5:**
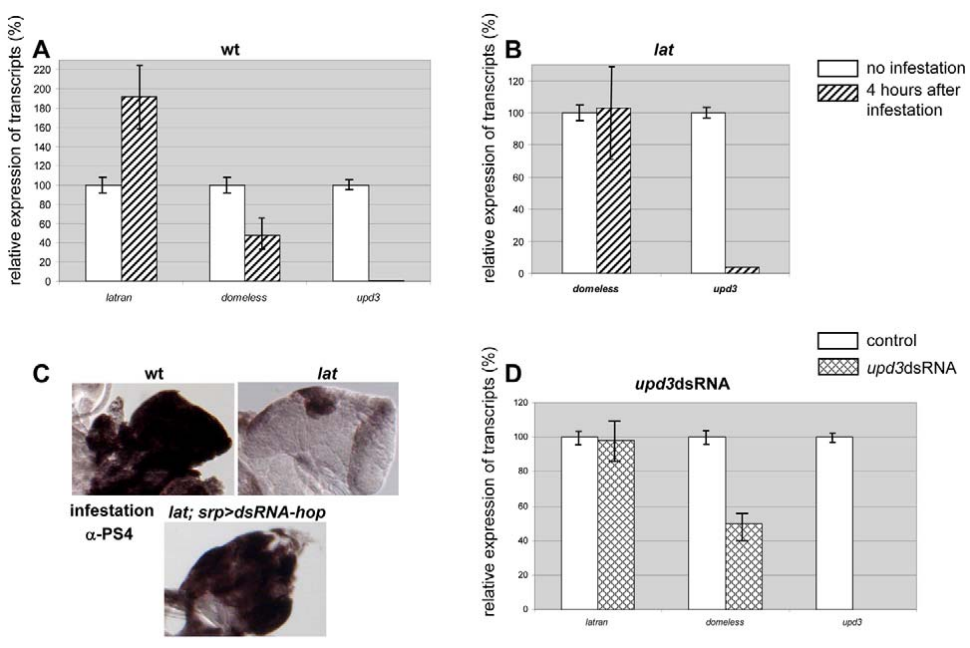
Downregulation of *upd3* and *dome* transcripts in response to wasp infestation. Quantitative analysis of *dome*, *lat*, and *upd3* relative to *rp49* transcripts is given in either (A) wt or (B) *lat* mutant LGs. A significant decrease of *dome* (*p* = 5×10^−5^) and increase of *lat* (*p* = 1.5×10^−16^) transcripts is observed 4 h after wasp egg-laying. The level of *upd3* transcripts drops to almost undetectable. This decrease of *upd3* mRNA is also observed in *lat* mutant LGs (B). (C) αPS4 (lamellocytes, black) immunostaining of wt, *lat* mutant, and *lat,srp>dsRNA-hop LGs* 30 h following wasp infestation. Massive lamellocyte differentiation is observed in *lat;srp>dsRNA-hop* mutant LGs similar to wt. (D) *dome*-Gal4>* upd3*dsRNA-induced degradation of *upd3* transcripts in the LG leads to a significant decrease of *dome* (*p* = 1×10^−8^) transcripts whereas the level of *lat* (*p* = 0.2) is not affected. *rp49* and *rpL17* mRNAs were used as internal controls (unpublished data). Three independent experiments were performed, vertical bars correspond to SD.

Both cell culture and in vivo data pointed to the Lat/Dome ratio as a key component in the regulation of JAK/STAT signalling. To determine whether this ratio is modified upon wasp parasitisation, we compared the levels of accumulation of *lat* and *dome* mRNAs in LGs relative to internal controls (*rp49* and *rpL17*, ribosomal protein mRNAs). Quantitative RT-PCR (qRT-PCR) measurements were performed on total LG RNA from control larvae and larvae 4 h after wasp egg-laying in order to mainly detect primary changes that occur in response to parasitism [48]. We detected an about 2-fold increase in *lat* transcripts and 2-fold decrease in *dome* transcripts, which results in a significant drop in the *dome/lat* ratio (Figure 5A). Since JAK/STAT signalling remains on after infestation in *lat* mutant larvae, we repeated the analysis on RNAs from *lat* mutant LGs. In this case, no decrease in the level of *dome* transcripts was observed upon wasp infestation, indicating that this decrease depends upon *lat* activity (Figure 5B). In order to strengthen this conclusion, we tested whether decreasing JAK/STAT signalling in the LG by expressing dsRNA-*hop* (*srp*-Gal4 > dsRNA-hop) could rescue the *lat* mutant phenotype. We indeed observed that lowering the level of Hop activity restored the ability of *lat* mutant LG to massively produce lamellocytes following wasp parasitism (Figure 5C), confirming that *lat* acts upstream of *hop* in the signalling cascade. Together, these data lead us to propose that the shift in the relative levels of *dome* and *lat* expression observed following wasp egg-laying operates as a switch leading to a complete extinction of JAK/STAT signalling, thus allowing prohemocytes to differentiate.

### *upd3* Is Expressed and Required for JAK/STAT Signalling Activity in the MZ

**Figure 6:**
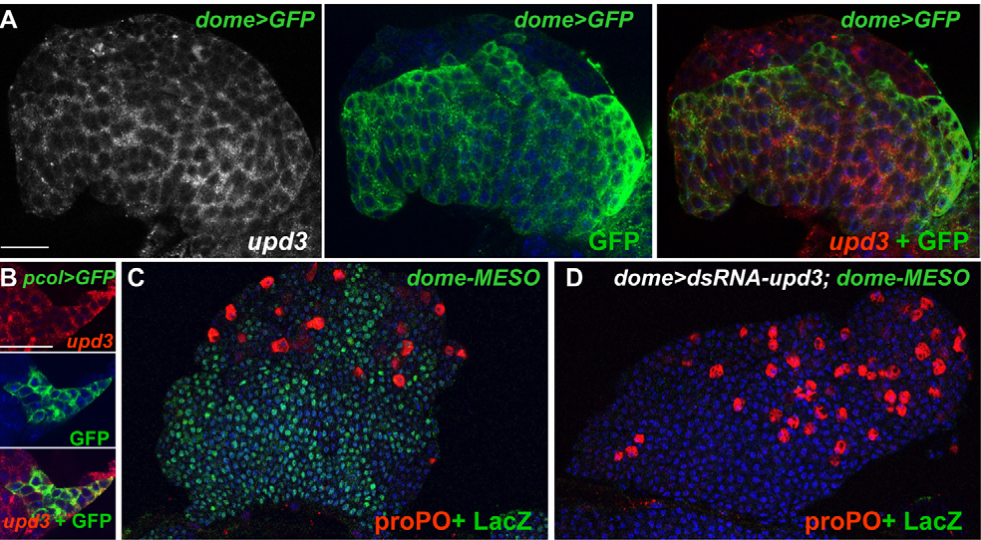
*upd3* is expressed and required to maintain JAK/STAT signalling in prohemocytes. (A) *upd3* expression (white and red, left and right panels, respectively) overlaps that of dome > GFP (green, middle and right panels) in LGs of third instar larvae. (B) *upd3* (red, top panel) is also expressed in the PSC (pcol>GFP, green, middle panel), overlay at the bottom. (C and D) dome-Gal4 driven expression of *upd3* dsRNA leads to loss of dome-MESO expression (LacZ, green) in the MZ. proPO staining (red) indicates differentiating crystal cells. Nuclei (TOPRO-3) are in blue. Scale bars, 40 µm (A); 60 µm (B).

Three different ligands, Upd, Upd2, and Upd3, are known to activate JAK/STAT signalling in *Drosophila* [47],[49]. In order to determine which of them are expressed in the LG, we first performed RT-PCR experiments, starting from LG mRNA. We detected *upd3* and very low amounts of *upd2* but no *upd* transcripts (Figures 5, 6, and S8). Since *upd2* mutants have no hematopoietic phenotype (unpublished data), *upd2* was not further considered in this study. We then focused on *upd3* expression and function. Since only genome annotation data were available, we determined the 5′ end of *upd3* transcript by RACE-PCR and repositioned the ATG initiation codon (Figure S8A). In situ hybridisation of *upd3* transcripts in Dome > GFP and pcol > GFP LGs indicated that *upd3* is expressed in the MZ, the PSC, and in few scattered cells of the CZ (Figure 6A, 6B). While a *upd3* loss of function mutant is not available, studies performed in vivo and in cell culture have established that *upd3* dsRNA expression can efficiently suppress *upd3* activity [50]. We looked at the consequence of *upd3* dsRNA expression (*UAS-upd3 dsRNA/dome-Gal4*), which drastically reduces *upd3* mRNA level in the LG (Figure 5D), on dome-MESO expression. No dome-MESO expression could be detected, showing that *upd3* expression in the MZ is required to maintain JAK/STAT signalling active (Figure 6C, 6D). When *upd3* dsRNA expression was targeted to the PSC (pcol-Gal4), dome-MESO expression was unperturbed (unpublished data). We then determined whether *upd3* levels are modified upon wasp parasitisation. The drastic decrease of *upd3* transcripts observed 4 h after infestation (Figure 5A) shows that *upd3* downregulation is an immediate response to wasp parasitisation.

**Figure 7:**
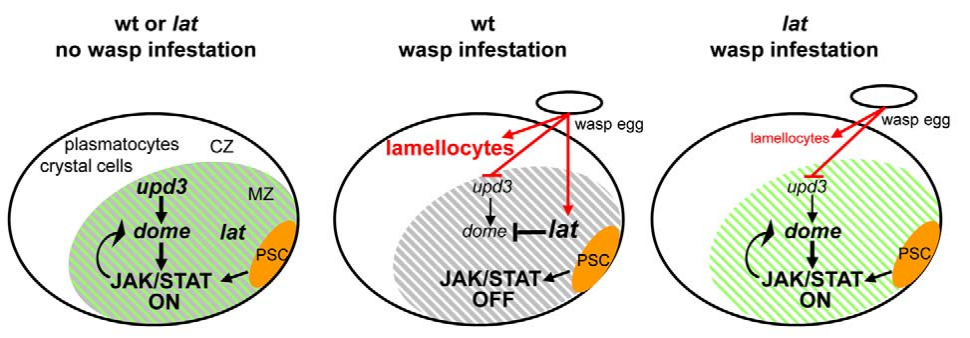
Model for *lat* function in *Drosophila* larval hematopoiesis. During normal development (left panel), PSC cells (orange) act, in a non–cell-autonomous manner (arrow) to maintain JAK/STAT signalling activity and preserve a pool of multipotent prohemocytes in the MZ (green shades). The PSC signal overrides *lat* function in the MZ (grey shades). In response to parasitisation (middle panel), there is a decrease of *upd3* and *dome* and increase of *lat* transcripts, which ultimately lead to an increased *lat/dome* ratio. The PSC signal is short-circuited. As a result, JAK/STAT signalling is switched off, thus licensing prohemocytes to differentiate into lamellocytes. Lat activity is strictly required in the LG for this switch. In the absence of *lat* (right panel), residual *upd3* levels maintain JAK/STAT activity, therefore preserving a pool of prohemocytes (grey shades). Upon wasp parasitisation some differentiating hemocytes become lamellocytes, however, indicating that *lat* is not required for this differentiation program per se. Arrows indicate activation, vertical bars repression.

While JAK/STAT signalling is dependent upon the binding of Upd to Dome, *dome* is itself a target of the JAK/STAT pathway in the embryonic mesoderm [36], a regulatory loop reproduced by the dome-MESO enhancer in the LG [9],[36]. To directly test whether the decreased amount of *dome* transcripts in the LG that follows wasp parasitisation could result from the drop of *upd3* activity, we measured the relative amounts of *dome* and *lat* transcripts upon *upd3 dsRNA* expression in the MZ. Whereas the *lat* level was not affected, a 2-fold decrease was observed for *dome* transcripts (Figure 5D). We conclude that the decrease in *dome* transcripts is a secondary response consecutive to decreased levels of *upd3* mRNA. Unlike *dome*, however, *upd3* downregulation is independent o*f lat* function (Figure 5B). Thus, we propose the following model: wasp parasitism results in a drastic decrease in *upd3* levels, which in turn leads to a downregulation of JAK/STAT signalling and a decrease of *dome* transcription. This, in turn, results in an increased *lat/dome* ratio, which subsequently leads to the complete shut down of the JAK/STAT pathway. The complete and efficient inhibition of JAK/STAT signalling in the LG thus requires *lat* function (Figure 7).

## Discussion

The evolutionarily conserved JAK/STAT signalling pathway was discovered from studies on the role of interferon in the control of immune responses. Vertebrate genomes encode multiple forms of all major JAK/STAT pathway components, including multiple receptor subunits. As opposed to this, in *Drosophila*, only one functional receptor, Dome, had so far been characterised. However, sequence similarity between *dome* and the neighbouring gene *CG14225/lat* suggested a gene duplication event and raised the question of *lat* function.

### Switching off JAK/STAT Signalling and Orienting Prohemocytes towards a Lamellocyte Fate: Two Facets of the *Drosophila* Immune Response to Wasp Parasitisation

In order to neutralise parasitoid wasp eggs, the *Drosophila* larval hematopoietic organ must rapidly release large amounts of lamellocytes in the hemolymph. In normal developmental conditions, the PSC maintains JAK/STAT signalling in the hematopoietic progenitor zone (MZ) thereby preserving their prohemocyte character. Upon wasp infestation, however, JAK/STAT signalling is switched off, leading to a concerted differentiation of prohemocytes into lamellocytes [9]. In *col* mutant LGs, which are devoid of PSC cells, no lamellocytes differentiate after wasp infestation as a consequence of the premature loss of multipotent prohemocytes; conversely, in *lat* mutant larvae, prohemocytes are maintained and very few lamellocytes differentiate. Prohemocytes persist in *lat;col* double mutant LGs, suggesting that a basal level of JAK/STAT signalling subsists in this mutant context, leading to a stochastic rather than global differentiation of prohemocytes. The comparison of *col*, *lat*, and *lat;col* mutant phenotypes, therefore, allows to conclude that *lat* functions as a switch. In normal developmental conditions, PSC activity overrides *lat* function in the MZ. Upon wasp infestation, PSC activity is short-circuited and *lat* plays a decisive role in completely silencing the JAK/STAT pathway in all prohemocytes. Hemocyte homeostasis in the LG thus relies on both extrinsic signals from the niche and intrinsic JAK/STAT activity in progenitor cells (Figure 7). In *lat* mutants, some lamellocytes differentiate following wasp parasitisation indicating that *lat* is not strictly required for the lamellocyte differentiation programme per se. Thus, switching off JAK/STAT signalling and orienting prohemocytes towards a lamellocyte fate are two distinct responses to wasp parasitisation (Figure 7).

### Differential Regulation of *lat* and *dome* Expression Warrants Inactivation of the JAK/STAT Pathway upon Wasp Infestation

In situ hybridisation and LG-targeted RNA interference experiments show that *upd3* is expressed and required in the MZ to maintain JAK/STAT activity in prohemocytes, therefore acting in an autocrine and/or paracrine manner, as previously reported for Upd in embryos [47],[51],[52]. The drastic decrease of *upd3* expression induced by wasp egg-laying is accompanied by a significant decrease in *dome* transcripts, showing that *dome* is both a component and a target of JAK/STAT signalling in the MZ (Figure 5), as previously documented in the embryonic mesoderm [36]. *lat* and *dome* mRNA levels are not, however, coregulated in response to parasitisation even though the two genes lie very close to each other on the chromosome, a tandem organisation conserved in other *Drosophila* species. The uncoupling between *dome* and *lat* expression results in an increased *lat/dome* ratio following wasp infestation, which is determinant for the ability of Lat to antagonise Dome activity. Comparative analyses of RNAs from wt and *lat* mutant LGs show that the primary component of the JAK/STAT pathway that is affected by wasp infestation is the level of *upd3* transcripts. Although we do not know yet how *upd3* is downregulated, it is tempting to speculate that it could be at a post-transcriptional level, similar to the importance of post-transcriptional regulation for cytokine levels in vertebrates (for review [53]). In summary, our results show that a primary immune response to wasp egg-laying is a strong decrease in *upd3* mRNA levels in the LG, which induces a downregulation of the JAK/STAT pathway, followed by a decrease of *dome* and increase of *lat* levels. This results in an increased *lat/dome* ratio that further and completely turns off the JAK/STAT pathway. Since in the absence of *lat* the decrease in *upd3* level does not completely switch off the JAK/STAT pathway. We conclude that *Lat* acts as a switch that is required for the total arrest of JAK/STAT signalling in hematopoietic progenitors in response to wasp parasitisation, a prerequisite to massive differentiation of lamellocytes and efficient immune response (Figure 7).

### *dome* and *lat*, a Pair of Duplicated Genes with Antagonistic Functions

Dome is related to the human GP130 and cognate GP130-like (GPL) signalling receptors, which form heteromeric complexes with short, nonsignalling receptors such as IL-6R or Oncostatin M receptor (OSM-R) to mediate signalling (Figure S9) [26],[54],[55]. *lat* encodes a short-type receptor that could either act as IL-6R and confer signalling specificity to Dome or as a dominant-negative receptor similar to what has been described ex vivo for short receptors such as GPL and IL13Rα2 [27]. Cell-culture and in vivo assays show that Lat antagonises Dome activity in a dose-dependent manner and forms heteromers with Dome thereby acting as a dominant-negative receptor. Altogether, these data suggest that, following parasitism, which leads to decreased cytokine levels, Lat blocks Dome activity in the LG through the formation of inactive heteromers.

While our analysis indicates that *lat* is specifically required in the larval hematopoietic organ for massive lamellocyte production in response to an immune challenge, phenotypes induced by ectopic *lat* expression show that it can antagonise JAK/STAT activity in other tissues. Together, the phenotypic and protein interaction data suggest that LG specific *lat* expression has been selected during evolution to fulfil specific immune functions.

Cytokine signalling pathways are subject to extensive positive and negative feedback regulations, which are crucial to generate appropriate physiological responses [22]. Two genome-wide RNA interference (RNAi) screens for JAK/STAT signalling components were conducted in *Drosophila* cultured cells. While they identified large sets of putative positive and negative regulators, they failed to detect *lat*, either because its expression level in cell culture is too low to be functional or because the *lat* dsRNA constructs used in these screens were not efficient enough [29],[30],[56]. *col/kn w*as identified in one of these RNAi screens, however, as a positive regulator acting downstream of Hop [30], suggesting another possible level of regulation of JAK/STAT signalling in the LG. Initial evidence for the involvement of JAK/STAT signalling in *Drosophila* cellular immunity came from the observation that a dominant gain-of-function mutation of the JAK kinase (*hop^Tum^*) provokes the apparition of lamellocytes and melanotic masses in the absence of wasp infection. This finding led to the conclusion that upregulation of JAK/STAT signalling triggers lamellocyte differentiation, which is in apparent contradiction with our present data [32]. Whether constitutive JAK/STAT signalling in differentiating hemocytes could instruct them to become lamellocytes remains an interesting possibility. Of note, a STAT target, *chimno*, was recently shown to be expressed at higher levels in differentiating CZ cells as compared to undifferentiated MZ cells [57]. Recent studies further suggest a dual role of Wg signalling in the maintenance of prohemocytes and PSC cells [58]. A tight control of ROS levels in the MZ is also required to maintain a pool of prohemocytes [59]. How these different signalling pathways are interconnected with JAK/STAT signalling in order to maintain hemocyte homeostasis in the LG are important questions to be addressed in the future.

### A Conserved Role for Dominant-Negative Short Receptors in the JAK/STAT Pathway?

The type I cytokine receptor family has considerably expanded in vertebrates [60]. This expansion results both from an increased number of receptor genes and from the generation of various protein isoforms that can act as either receptors or coreceptors [61]. Soluble versions of short receptors isolated from diverse body fluids have also been identified, which behave as antagonists by competing with membrane-associated receptors for ligand binding [62],[63]. These soluble receptors are generated by either limited proteolysis or translation from differently spliced mRNAs. Finally, studies on IL13Rα2 [27] or GPL [64] suggested that short receptors anchored to the membrane could also behave as dominant negative receptors. However, the exact function of these receptors and how their expression is regulated and linked in vivo to tissue homeostasis, remains unknown. Our studies in *Drosophila* indicate that Lat acts as a dominant-negative receptor rather than a coreceptor, extending in vivo the few observations made in mammalian cell cultures [65],[66]. Tissue-specific regulation of JAK/STAT signalling in response to environmental cues is crucial for the ability of *Drosophila* to mount a cellular immune defense. Our results bring to light a new mode of fine tuning of the JAK/STAT pathway, that is, differential expression of signalling and antagonist cognate receptors. Whether and when regulated expression of long and short receptor isoforms is employed in controlling specific aspects of immunity in vertebrates certainly deserves further investigation.

## Materials and Methods

### *Drosophila* Strains

The following strains were used: *pcol85*-Gal4;UASmcd8GFP (pcol>GFP) [9]; PG125*-dome*-Gal4 (*dome*-Gal4) [67] and *srp*-Gal4 [11]. *ey*-Gal4 and *da*-Gal4 were obtained from the Bloomington *Drosophila* Stock Center. The dome-MESO, UAS-*dome*, UAS-DomeΔα, and UAS-DomeΔω strains are from [42]; the UAS-upd3dsRNA from [50]; and the P[70FLP][70I-SceI)/TM3 and P[ry+; FLP)10 (Chromosome II) from F. Karch. *white* strains were used as wt. Mid-second instar larvae subjected for 30–60 min to egg-laying by *Leptopilina boulardi* (G464) were allowed to develop at the appropriate temperature and analysed 4, 24, or 48 h later.

### Generation of a *lat* Nul Mutant by Site-Directed Recombination

The procedure was adapted from the Ends out Knock Out method [33]. A *lat* KO “donor” transgene was constructed in pW25 [68] by inserting 4 kb of 5′ and of 3′ flanking sequences of the *lat* gene separated by the mini-*white* gene and used to transform *white* mutant flies (). Two different inserts on the second chromosome were selected for the recombination-targeting protocol. Several independent *lat* KO lines were obtained and verified for deletion of *lat* and insertion of mini-*white* by PCR and Southern blot analyses (Figure S1C). The *lat^18A^* line was chosen for all the experiments.

### Constructs

The mapping of *lat* and *upd3* transcript 5′ ends was performed by 5′ RACE PCR (Marathon cDNA amplification kit, Clontech, and BD Smart RACE kit, BD Biosciences), using either polyA+ RNA from *hop^Tum-l^* larvae or total RNA from dissected *w* LGs. A full length *lat* cDNA was reconstructed and inserted in pUAS-T to generate UAS-Lat transgenic lines. UAS-LatΔα and UAS-LatΔω were constructed using the complete *lat* cDNA fused to the β-galactosidase Δα and Δω fragments from pUAS-Dome-LacZΔα and pUAS-Dome-LacZΔω, respectively [42]. The fusion constructs were subcloned into pUAS-attB to generate transgenic flies using the ZH49B and ZH86F attP integration platforms [69]. Act-Lat, Act-HALat, and Act-DomeV5 plasmids were constructed and used for cell culture experiments.

### RNA Probes

*A* 526-bp *lat* genomic fragment amplified using primers 6 and 8 (Figure S1A) was cloned in the Invitrogen pCRBluntII-TOPO vector. Two different *upd3* probes of 836 bp (primers 1 and 2) and 2,057 bp (primers 5 and 6) were designed for in situ hybridisation (Figure S8).

### In Situ Hybridisation, Antibody Staining, and Western Blotting

Dissections, in situ hybridisation, and immunostaining procedures were as described in [9],[70]. The following antibodies were used: rabbit anti-GFP (Torrey) 1/500; mouse anti-β-galactosidase (Promega) 1/800; rabbit anti-proPO 1/200; mouse anti-Col 1/50; rabbit anti-αPS4 [9] 1/200; mouse anti-V5 (Invitrogen) 1/5,000; mouse and rabbit anti-HA (Covance and Santa Cruz, respectively) 1/1,000. Mounting in Vectashield medium (Vector Laboratories) preceded analysis by confocal microscopy (Leica SP2).

### X-Gal Staining

X-Gal staining was as described in [42].

### RNA Amplification and Quantitative RT-PCR

Dissected LGs were collected in trizol and total RNA was extracted using trizol reagent (Invitrogen) according to the manufacturer. Superscript Reverse Transcriptase II (Invitrogen) and oligo dT primers were used for reverse transcription. Real-time quantitative PCR was performed on a MyiQ single color real time PCR detection system (Biorad). CT values were collected and analysis was performed according to the 2^ΔΔCT^ method [71] using *rp49* and *rpL17A* to normalize estimates of relative expression. Primers used: 1 and 3 and 5 and 7 for *dome* and *lat*, respectively (Figure S1), 3 and 4 for *upd3* (Figure S8). No significant differences were detected in the level of control RNAs in wt, *lat and dome > upd3dsRNA* experiments (Figure 5). Primers sequences for *rp49*, *rpL17A*, *upd*, and *upd2* are available on request. All qRT-PCR data are representative of three independent experiments and presented as means ± standard deviation (SD). Statistical analyses were performed using Student's *t* test.

### Cell Culture Experiments

Various amounts of Act-Lat, 0.2 ng of Act-Dome, and 1 ng of either Act-Upd, Upd2, or Upd3 were used to transfect S2-NP cells [29]. Luciferase assays were performed 4 d later, and the reporter activity was normalised as the ratio of firefly luciferase/Renilla. The results are from three independent experiments. For immunostaining, S2-NP cells were transfected with 1 ng of Act-upd and 0.2 ng of Act-Dome-V5 with or without 1 ng of Act-HALat (Figure 3E and 3F).

### Immunoprecipitation of HALat/DomeV5 Complex

*Drosophila* S2-NP cells [29] were maintained in Schneider medium +10% FCS + penicillin-streptomycin (Sigma 1/100) at 25°C without supplemental CO2. Cells (3×10^6^ per well) were seeded and cultured in six-well plates. 24 h later, transfections using Effectene (Quiagen) were performed. Each well was transfected with 20 ng of plasmid encoding either HA-Lat, Dome-V5, or both, and completed with plasmid DNA encoding the empty vector (pHA vector) to a final amount of 400 ng of DNA. 48 h later, cells from each well were washed in PBS and lysed in 150 µl of ice-cold buffer containing 50 mmol Tris (pH 7.4), 150 mmol NaCl, 1 mmol EDTA, 1% NP40, and antiprotease cocktail (Roche) for 20 min. 140 µl of the crude lysate was used for IP. Protein G sepharose beads (Sigma) were first incubated with 1 µg of anti-HA or anti-V5 antibodies for 1 h at 4°C and then with the cleared supernatant for 2 h at 4°C. Beads were then boiled in denaturing sample buffer and the released proteins loaded on a gel with 3 µl of the crude lysate (1/50 of the total preparation) used as a control lane. The separated proteins were analysed by Western blotting with either mouse anti-V5 or mouse anti-HA antibodies.

## Supporting Information

Figure S1 **The** ***D. melanogaster dome/lat/zw*** **genomic region.** (A) Nucleotide sequence of the *D. melanogaster dome/lat/zw* genomic region between the *dome* and *zw* transcription starts, as extracted from Flybase. Vertical arrows in the margin indicate the direction of transcription. ORFs are in bold capital letters, untranslated 5′ and 3′ sequences are in bold italic lower case, introns and intergenic regions are in lower case. Transcription starts are indicated by an arrowhead with +1, translation initiation codons (ATG) are underlined, and stop codons are circled. Primers used for PCR and RT-PCR experiments are underlined and numbered. Note that the position of the *lat* ATG differs from that found in FlyBase. The genomic region deleted by homologous recombination in *lat* mutant is labelled in yellow. The dashed line indicates the DNA fragment used to detect the *lat* sequence on Southern blots (see below). (B) Schematic of the donor DNA fragment used to generate a *lat* KO. Top line, *lat* genomic structure, (see Figure 1A); bottom, *lat* KO transgene, with the positions of primers, as indicated in (A). (C) Southern blot analysis of genomic DNA from three independent *lat* KO strains (*18A*, *18C*, *and 21D*) and controls. Position of the *lat* probe is indicated (Figure S1A, dashed line). In contrast to control flies, no DNA fragments corresponding to *lat* were detected in *lat* mutants, whereas two separate fragments were detected for *white*, confirming the insertion of the mini*-whit*e gene.
(0.43 MB DOC)

Figure S2 **Sequence alignment of the** ***D. melanogaster*** **Dome and Lat proteins.** ClustalW alignment of the Dome and Lat amino-acid sequences (http://npsapbil.ibcp.fr/cgibin/npsa_automat.pl?page=/NPSA/npsa_clustalw.html). The CBM (green letters, the signature of the motif is in bold), LDHR (blue), fibronectin repeats (red), transmembrane domain (bold), and STAT binding site (purple) are indicated. Stars and points indicate identical and similar amino acids, respectively. Black arrowheads indicate the position of introns.
(0.71 MB DOC)

Figure S3 **An evolutionary dendogram of Dome and Lat.** Search for *dome/lat* related genes was based on blast analyses using either the CBM region or the entire protein sequences. Complete amino-acid sequences encoded by each gene were compared with ClustalW. The dendogram was drawn, on the basis of the CBM sequence using the Phylip-Neighbor program (http://toolkit.tuebingen.mpg.de/sections/classification). Species abbreviations: Dmel (*D. melanogaster*), Dyak (*D. yakuba*), Dana (*D. ananassae*), Dmoja (*D. mojavensis*), Dpse (*D. pseudoobscura*), Dvir (*D. virilis*). Scale bar represents the number of substitution/site.
(1.07 MB TIF)

Figure S4 **Lat is not required for the ontogeny of the LGs and differentiation of plasmatocytes and crystal cells under nonimmune conditions.** The MZ and PSC develop in *lat* mutant LGs (B, D, F, H, J) as in wild type (A, C, E, G, I), as visualised by *tep4* (A, B), LacZ (dome-MESO, C, D) and *col* (E, F), respectively. Differentiating plasmatocytes (P1, H) and crystal cells (*doxA3*, J) are found in the CZ (G, I).
(2.51 MB TIF)

Figure S5 **Stochastic differentiation of hemocytes in** ***lat;col*** **double mutant LG.** (A, B) Intermingling of prohemocytes (green) and differentiating hemocytes, here crystal cells expressing proPO (red), is observed in *lat;col* double mutant LGs; (I) some lamellocytes differentiate following wasp egg-laying (integrin α chain [α-PS4], red arrow). Nuclei (TOPRO-3) are in blue.
(1.45 MB TIF)

Figure S6 ***lat*** **negatively regulates the JAK/STAT pathway.** *Drosophila* S2-NP cells were transfected with 10×STAT92E-luciferase, Act-*Renilla* and 1 ng of either Act-*upd* (*upd*), *Act-upd2* (*upd2*, top panel), or *Act upd3* (*upd3*, bottom panel) together with 0.2 ng of either *Act-Dome* or *Act-Lat*. Luciferase assays were performed 4 d later, and the reporter activity was normalised as the ratio of Firefly luciferase/Renilla. The results are from three independent experiments. Vertical bars correspond to SD.
(0.26 MB TIF)

Figure S7 ***lat*** **antagonises the JAK/STAT pathway in the eye.** *eyeless* (*ey*)*-Gal4* driven expression of *lat* in the eye disc leads to significant reduction of the eye. Flies were raised at 29°C.
(0.78 MB TIF)

Figure S8 **Mapping the** ***upd3*** **transcription start and initiation codon.** (A) Nucleotide sequence of the *D. melanogaster upd3* genomic region as extracted from Flybase. ORFs are in bold capital letters, untranslated 5′ and 3′ sequences in bold italic lower case, introns and intergenic regions in lower case. Since only genome annotation data were available for *upd3*, we verified the 5′ end of *upd3* by RACE-PCR, starting from total RNA isolated from larval LGs. The transcription start is indicated by an arrowhead with +1, the translation initiation codon (ATG) underlined, and the stop codon circled. Primers used are underlined and numbered. (B) RT-PCR analysis of *upd*, *upd2*, and *upd3* expression in LGs. Left, PCR amplification on control genomic DNA; right, PCR amplification from RNA of dissected LG. Only *upd3* expression is detected at significant levels.
(2.16 MB DOC)

Figure S9 **Schematic of type I cytokine receptors from** ***Drosophila*** **and Vertebrates.** The green box corresponds to the CBM and the blue box to the LDHR; Fibronectin III (FnIII) motifs are indicated in red; the signal peptide in yellow. TM indicates the transmembrane domain. The intracytoplasmic regions are in grey, with the position of the STAT and JAK binding sites in orange and black, respectively. Ig-like domains are highlighted by circles.
(0.21 MB TIF)

## Abbreviations

CBM: cytokine binding motif
Col: Collier
CZ: cortical zone
Dome: Domeless
GFP: green fluorescent protein
JAK/STAT: janus tyrosine kinase/signal transducers and activators of transcription
Lat: Latran
LDHR: Lat-Dome homology region
LG: lymph gland
MZ: medullary zone
proPo: prophenoloxidase
PSC: posterior signalling centre
RT PCR: reverse transcriptase PCR
SD: standard deviation
Upd: Unpaired
wt: wild type
