# Supplementary figures and images for: A Short Receptor Downregulates JAK/STAT Signalling to Control the Drosophila Cellular Immune Response
Source: PLoS Biol. 2010 Aug 3;8(8):e1000441. doi: 10.1371/journal.pbio.1000441 (PMC2914635; doi:10.1371/journal.pbio.1000441)

| 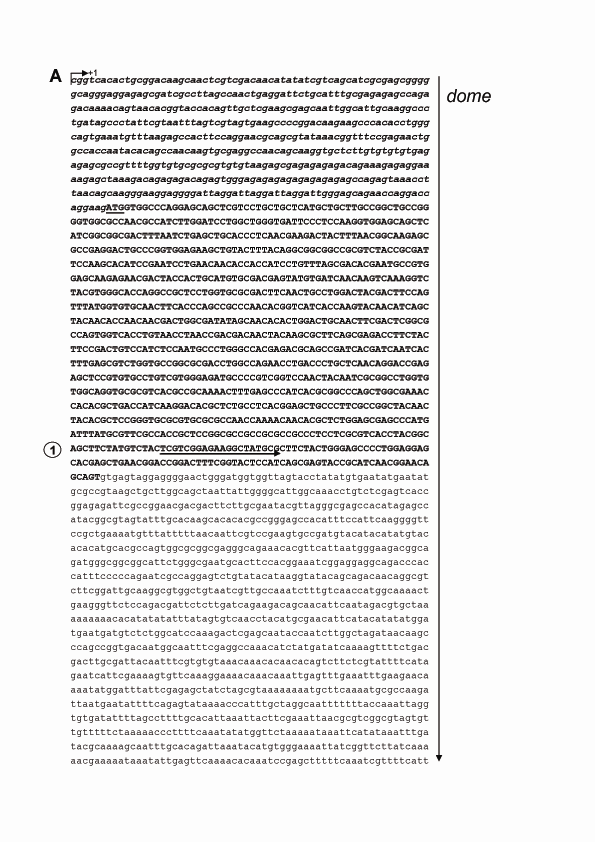 |
| --- |

| 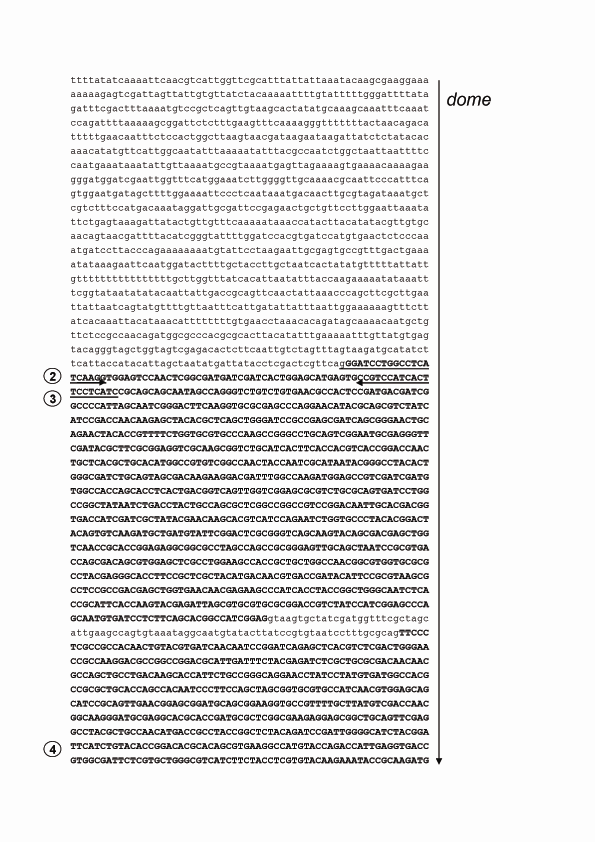 |
| --- |

| 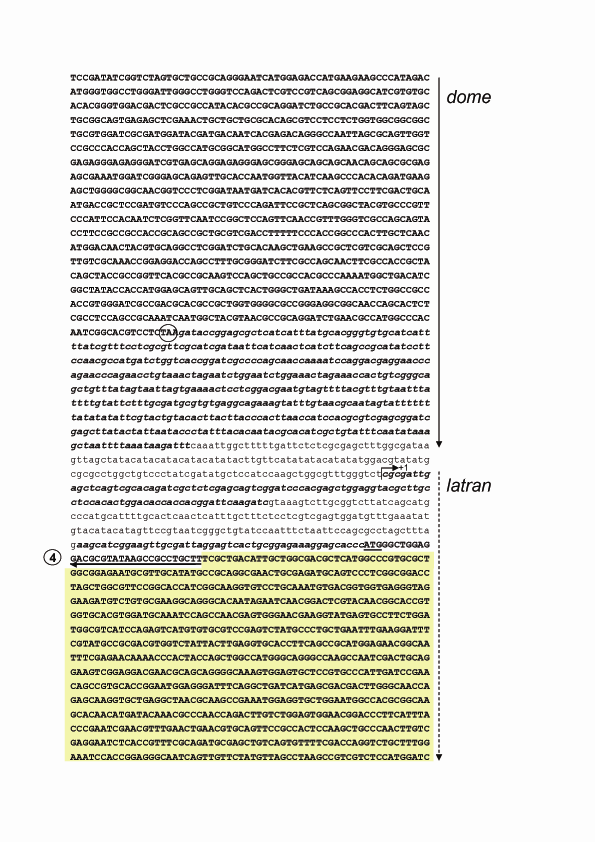 |
| --- |

| 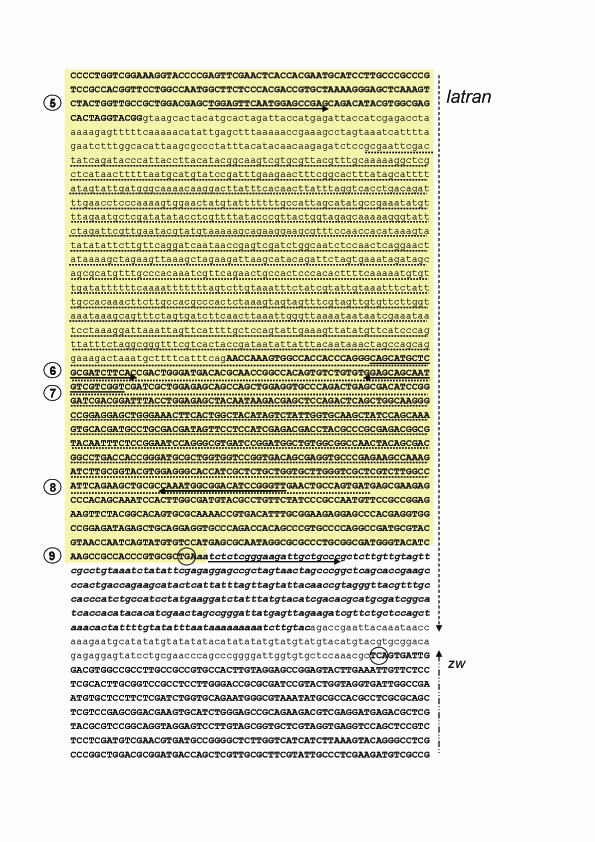 |
| --- |
| 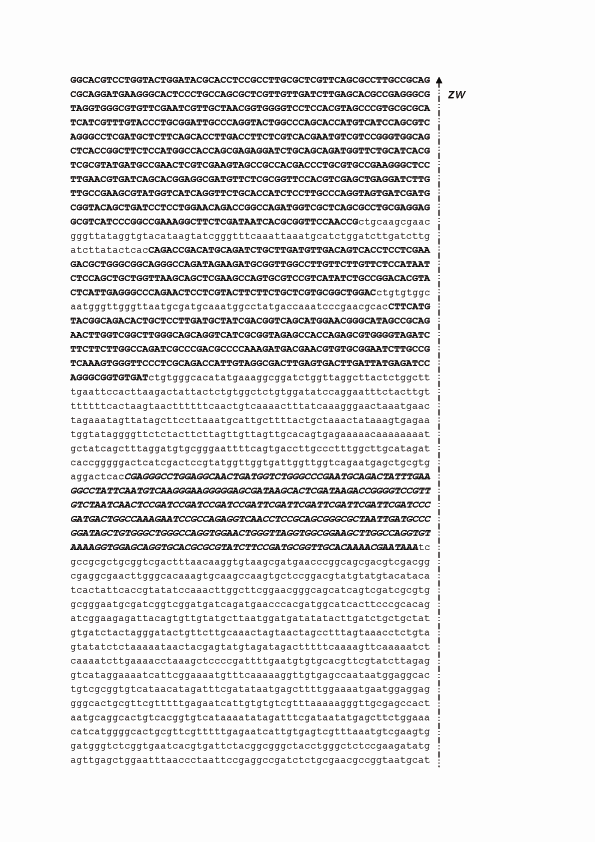 |

| 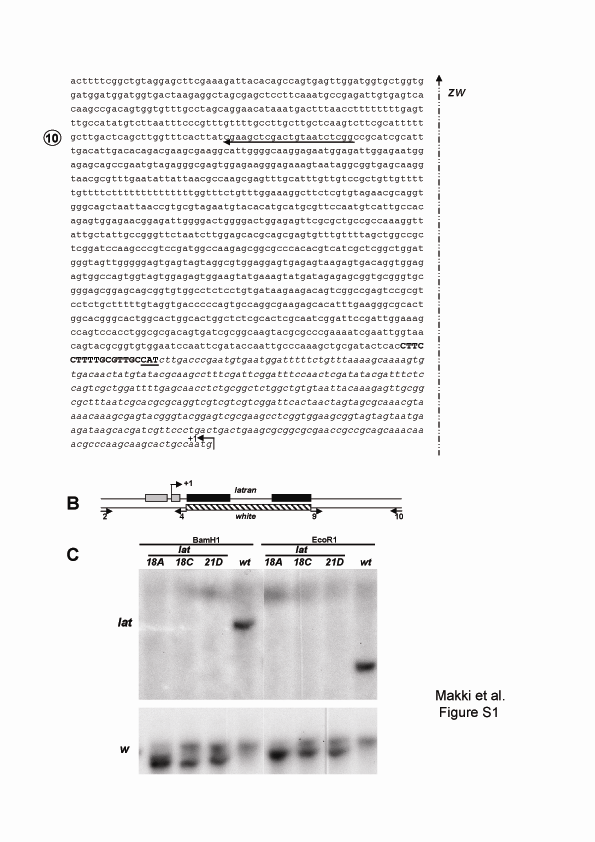 |
| --- |

Supplement: Figure S1 — The D. melanogaster dome/lat/zw genomic region. (A) Nucleotide sequence of the D. melanogaster dome/lat/zw genomic region between the dome and zw transcription starts, as extracted from Flybase. Vertical arrows in the margin indicate the direction of transcription. ORFs are in bold capital letters, untranslated 5′ and 3′ sequences are in bold italic lower case, introns and intergenic regions are in lower case. Transcription starts are indicated by an arrowhead with +1, translation initiation codons (ATG) are underlined, and stop codons are circled. Primers used for PCR and RT-PCR experiments are underlined and numbered. Note that the position of the lat ATG differs from that found in FlyBase. The genomic region deleted by homologous recombination in lat mutant is labelled in yellow. The dashed line indicates the DNA fragment used to detect the lat sequence on Southern blots (see below). (B) Schematic of the donor DNA fragment used to generate a lat KO. Top line, lat genomic structure, (see Figure 1A); bottom, lat KO transgene, with the positions of primers, as indicated in (A). (C) Southern blot analysis of genomic DNA from three independent lat KO strains (18A, 18C, and 21D) and controls. Position of the lat probe is indicated (Figure S1A, dashed line). In contrast to control flies, no DNA fragments corresponding to lat were detected in lat mutants, whereas two separate fragments were detected for white, confirming the insertion of the mini-white gene. (0.43 MB DOC) [file pbio.1000441.s001.doc]

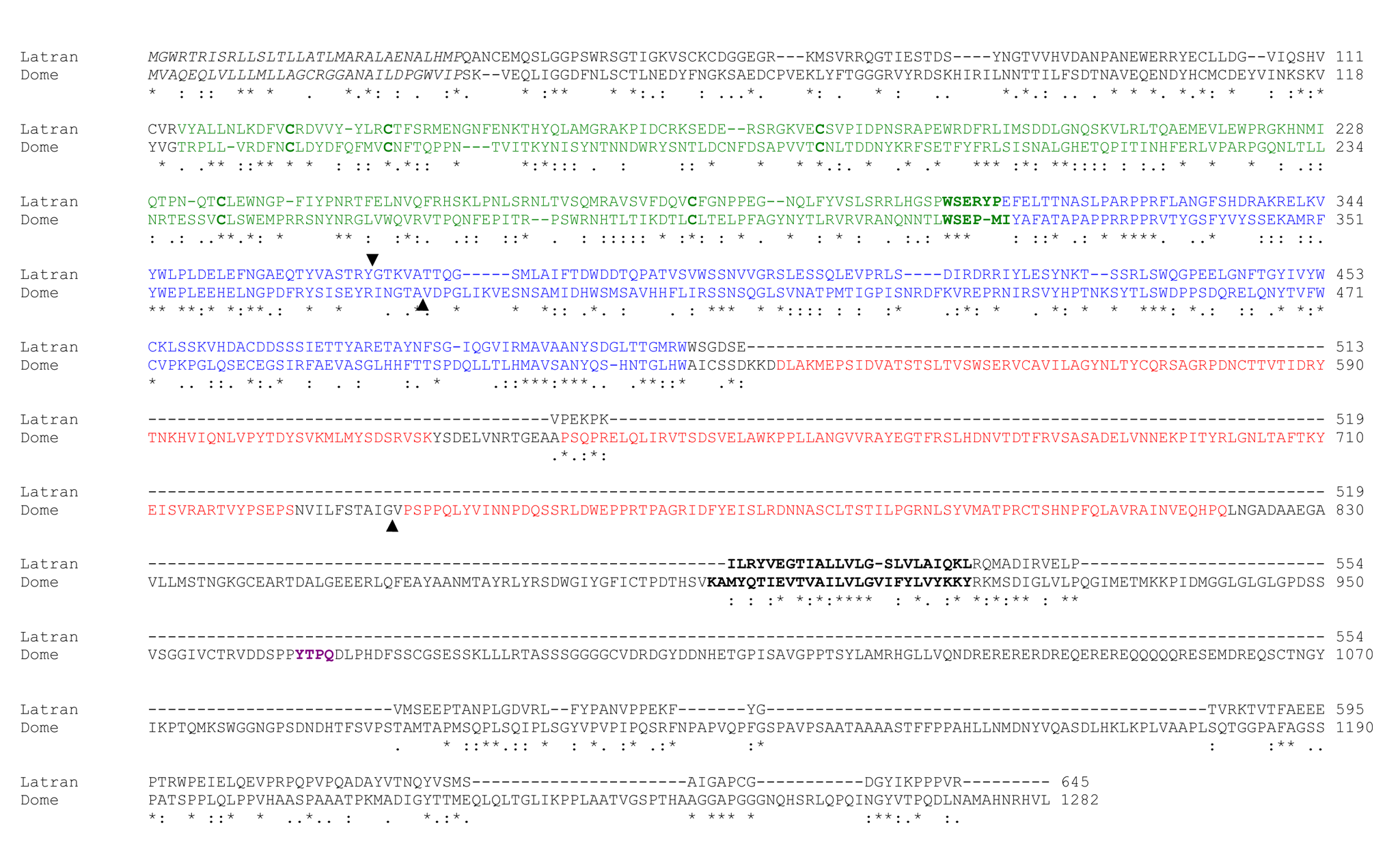

Supplement: Figure S2 — Sequence alignment of the D. melanogaster Dome and Lat proteins. ClustalW alignment of the Dome and Lat amino-acid sequences (http://npsapbil.ibcp.fr/cgibin/npsa_automat.pl?page=/NPSA/npsa_clustalw.html). The CBM (green letters, the signature of the motif is in bold), LDHR (blue), fibronectin repeats (red), transmembrane domain (bold), and STAT binding site (purple) are indicated. Stars and points indicate identical and similar amino acids, respectively. Black arrowheads indicate the position of introns. (0.71 MB DOC) [file pbio.1000441.s002.tif]

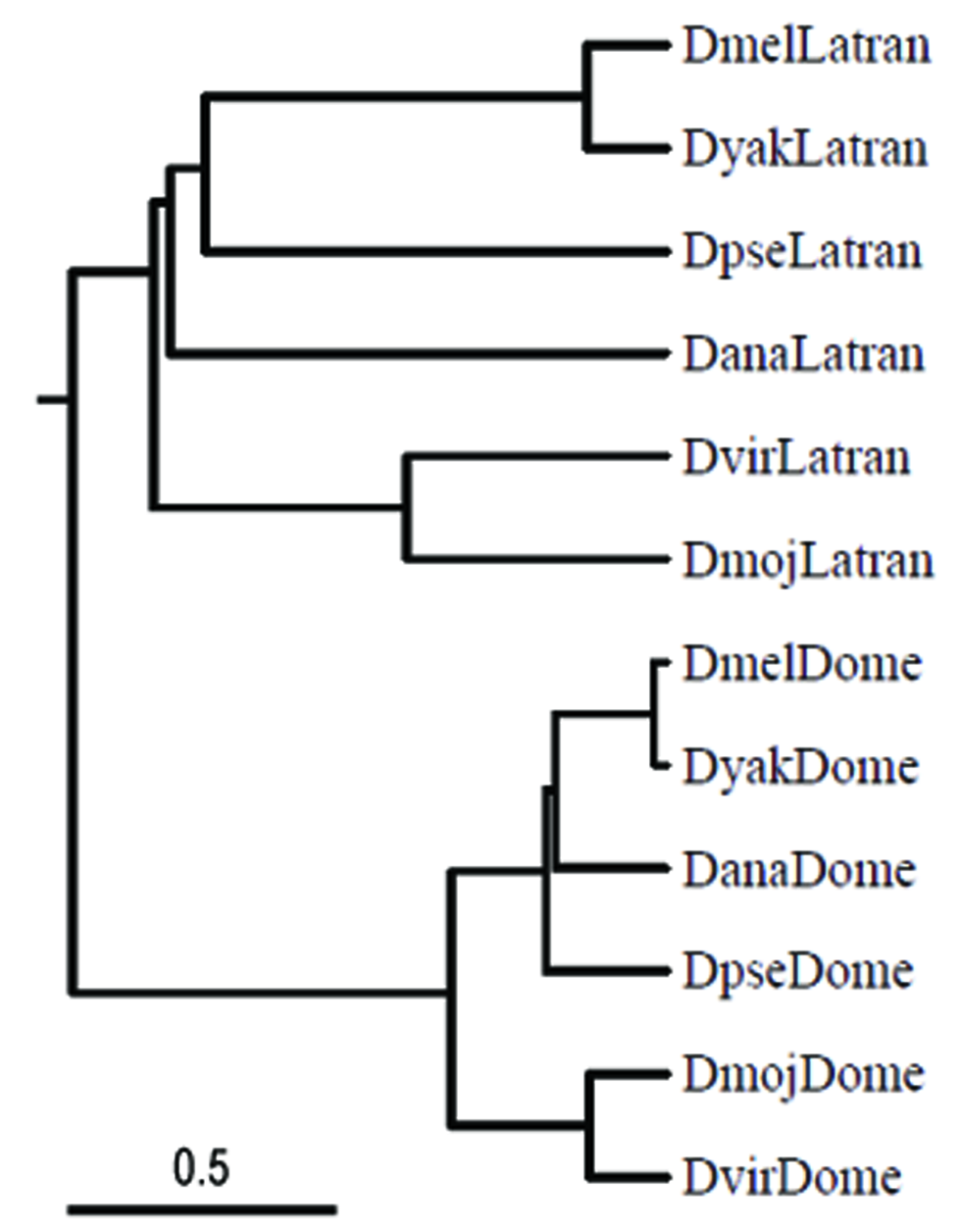

Supplement: Figure S3 — An evolutionary dendogram of Dome and Lat. Search for dome/lat related genes was based on blast analyses using either the CBM region or the entire protein sequences. Complete amino-acid sequences encoded by each gene were compared with ClustalW. The dendogram was drawn, on the basis of the CBM sequence using the Phylip-Neighbor program (http://toolkit.tuebingen.mpg.de/sections/classification). Species abbreviations: Dmel (D. melanogaster), Dyak (D. yakuba), Dana (D. ananassae), Dmoja (D. mojavensis), Dpse (D. pseudoobscura), Dvir (D. virilis). Scale bar represents the number of substitution/site. (1.07 MB TIF) [file pbio.1000441.s003.tif]

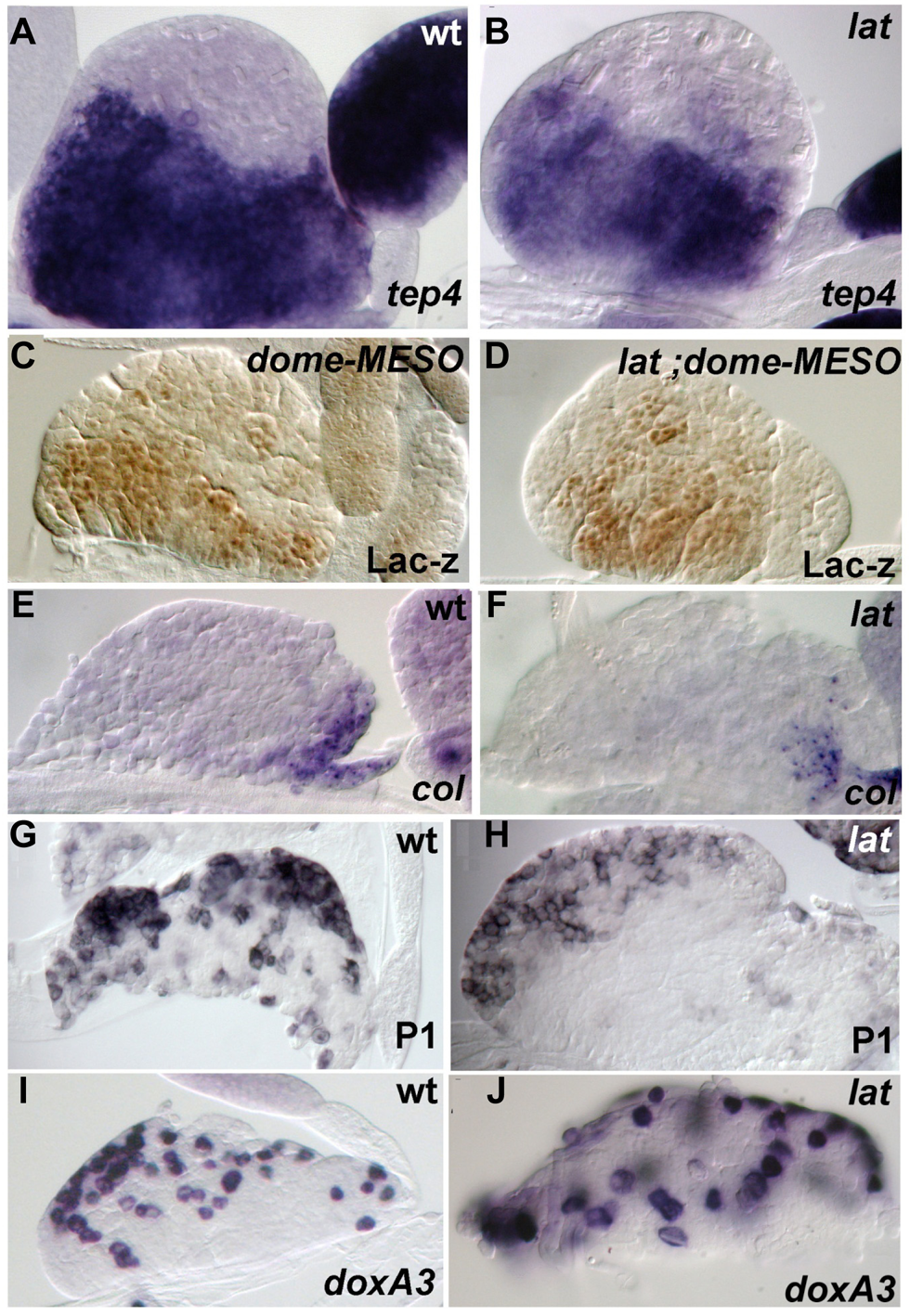

Supplement: Figure S4 — Lat is not required for the ontogeny of the LGs and differentiation of plasmatocytes and crystal cells under nonimmune conditions. The MZ and PSC develop in lat mutant LGs (B, D, F, H, J) as in wild type (A, C, E, G, I), as visualised by tep4 (A, B), LacZ (dome-MESO, C, D) and col (E, F), respectively. Differentiating plasmatocytes (P1, H) and crystal cells (doxA3, J) are found in the CZ (G, I). (2.51 MB TIF) [file pbio.1000441.s004.tif]

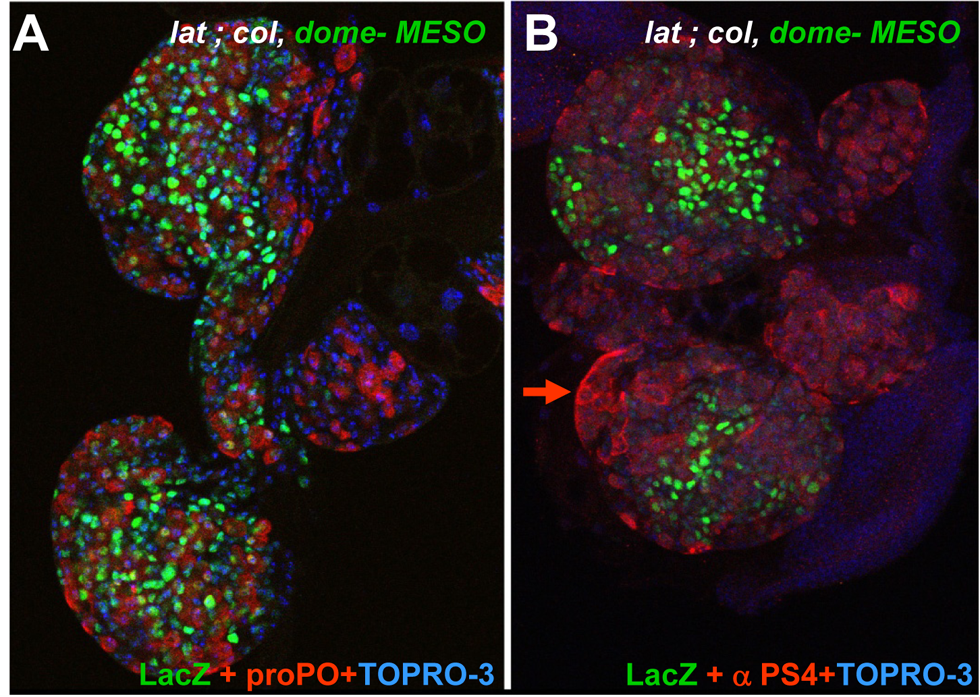

Supplement: Figure S5 — Stochastic differentiation of hemocytes in lat;col double mutant LG. (A, B) Intermingling of prohemocytes (green) and differentiating hemocytes, here crystal cells expressing proPO (red), is observed in lat;col double mutant LGs; (I) some lamellocytes differentiate following wasp egg-laying (integrin α chain [α-PS4], red arrow). Nuclei (TOPRO-3) are in blue. (1.45 MB TIF) [file pbio.1000441.s005.tif]

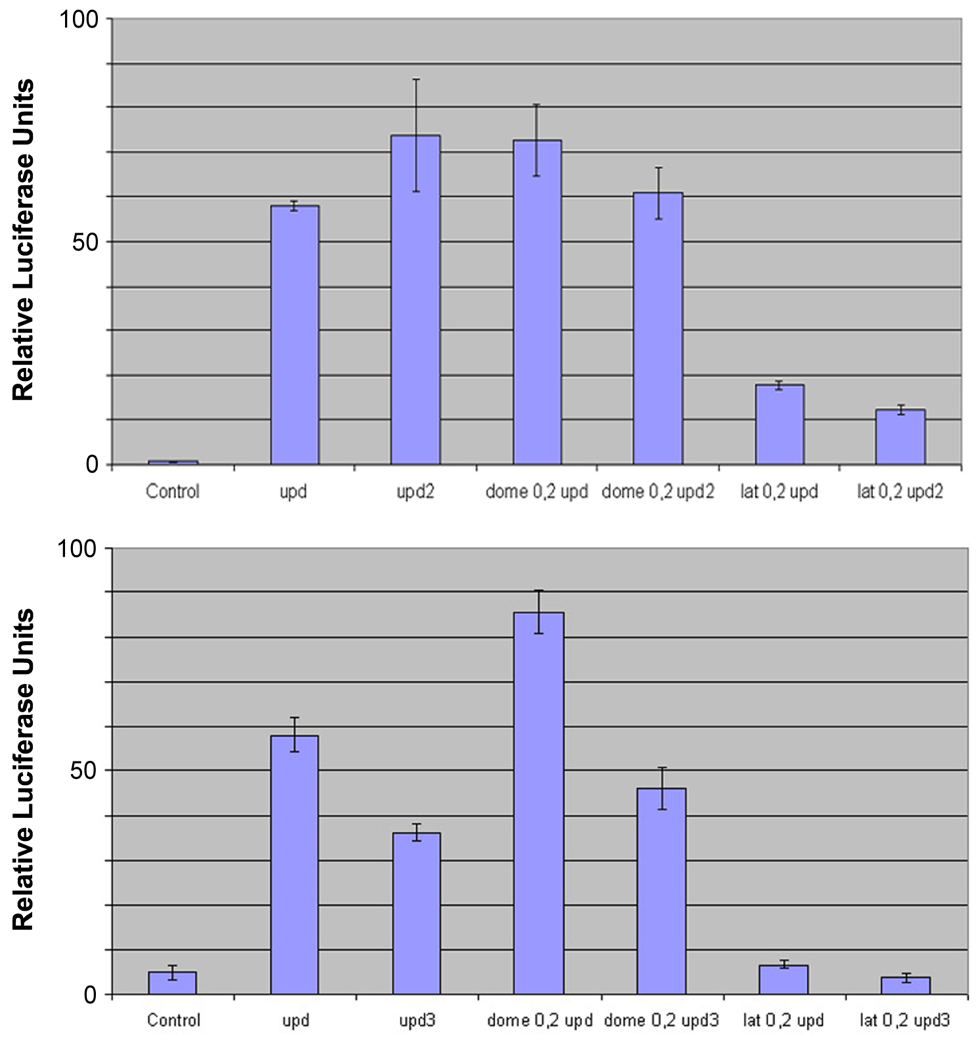

Supplement: Figure S6 — lat negatively regulates the JAK/STAT pathway. Drosophila S2-NP cells were transfected with 10×STAT92E-luciferase, Act-Renilla and 1 ng of either Act-upd (upd), Act-upd2 (upd2, top panel), or Act upd3 (upd3, bottom panel) together with 0.2 ng of either Act-Dome or Act-Lat. Luciferase assays were performed 4 d later, and the reporter activity was normalised as the ratio of Firefly luciferase/Renilla. The results are from three independent experiments. Vertical bars correspond to SD. (0.26 MB TIF) [file pbio.1000441.s006.tif]

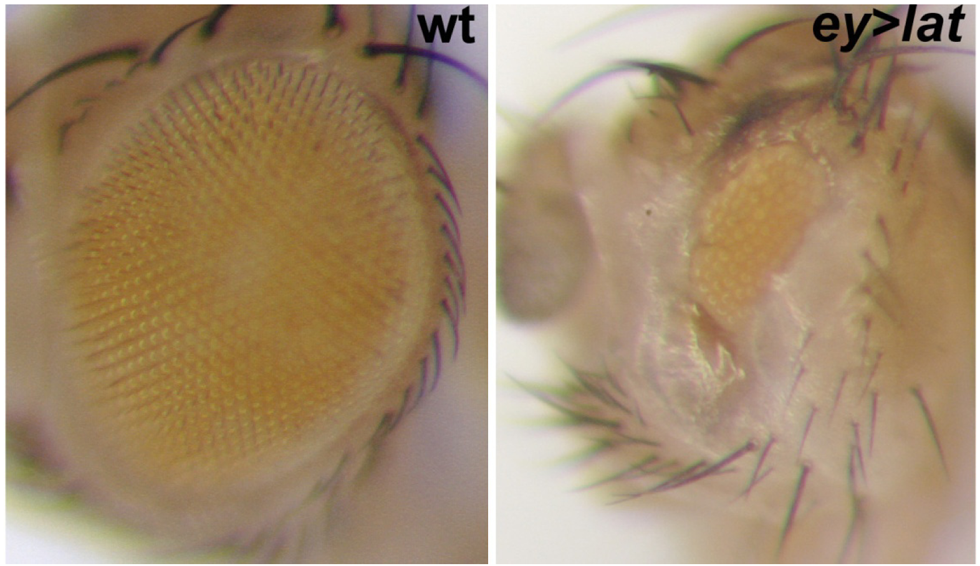

Supplement: Figure S7 — lat antagonises the JAK/STAT pathway in the eye. eyeless (ey)-Gal4 driven expression of lat in the eye disc leads to significant reduction of the eye. Flies were raised at 29°C. (0.78 MB TIF) [file pbio.1000441.s007.tif]

| | 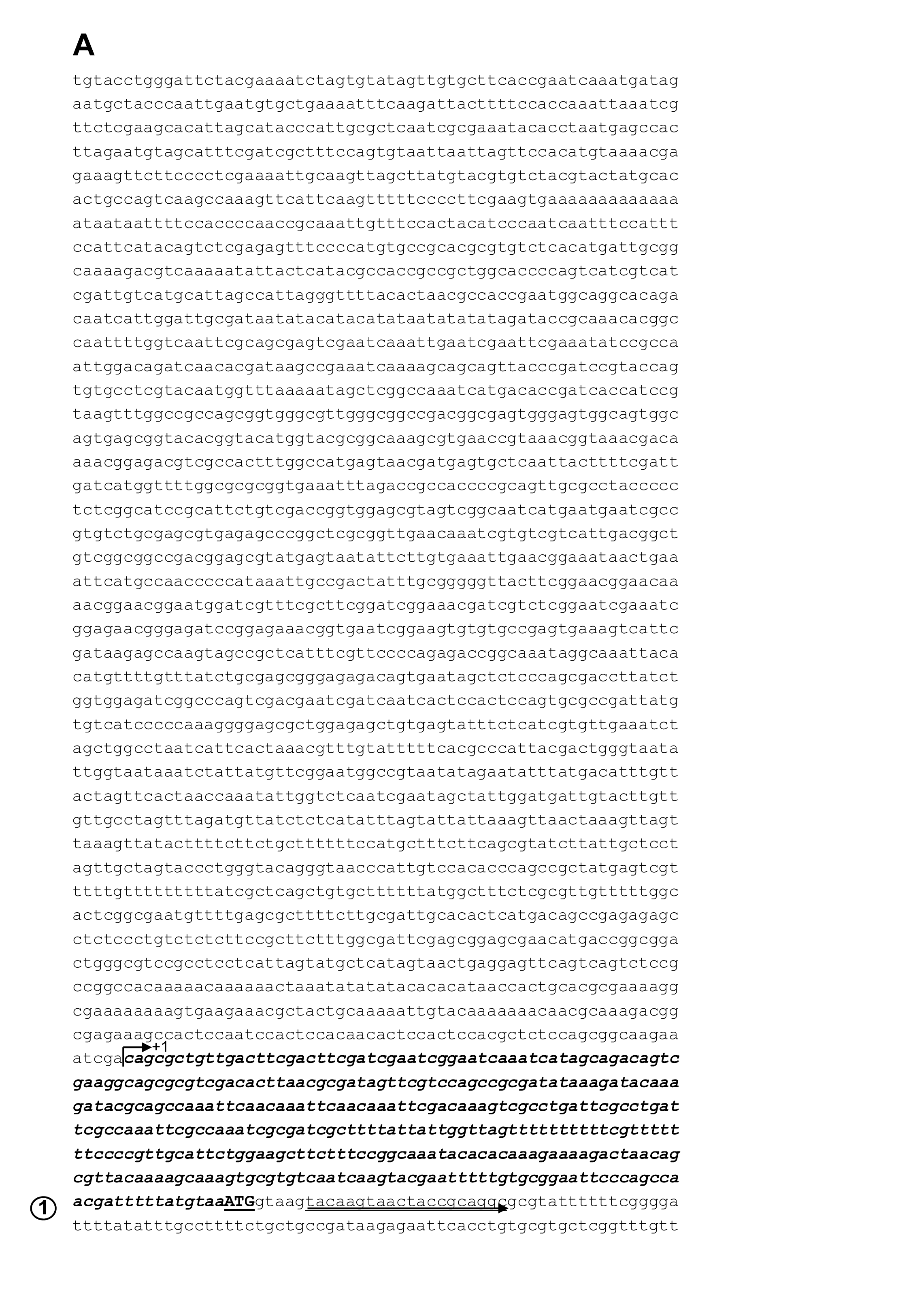 | | --- | |
| --- | --- |
| | 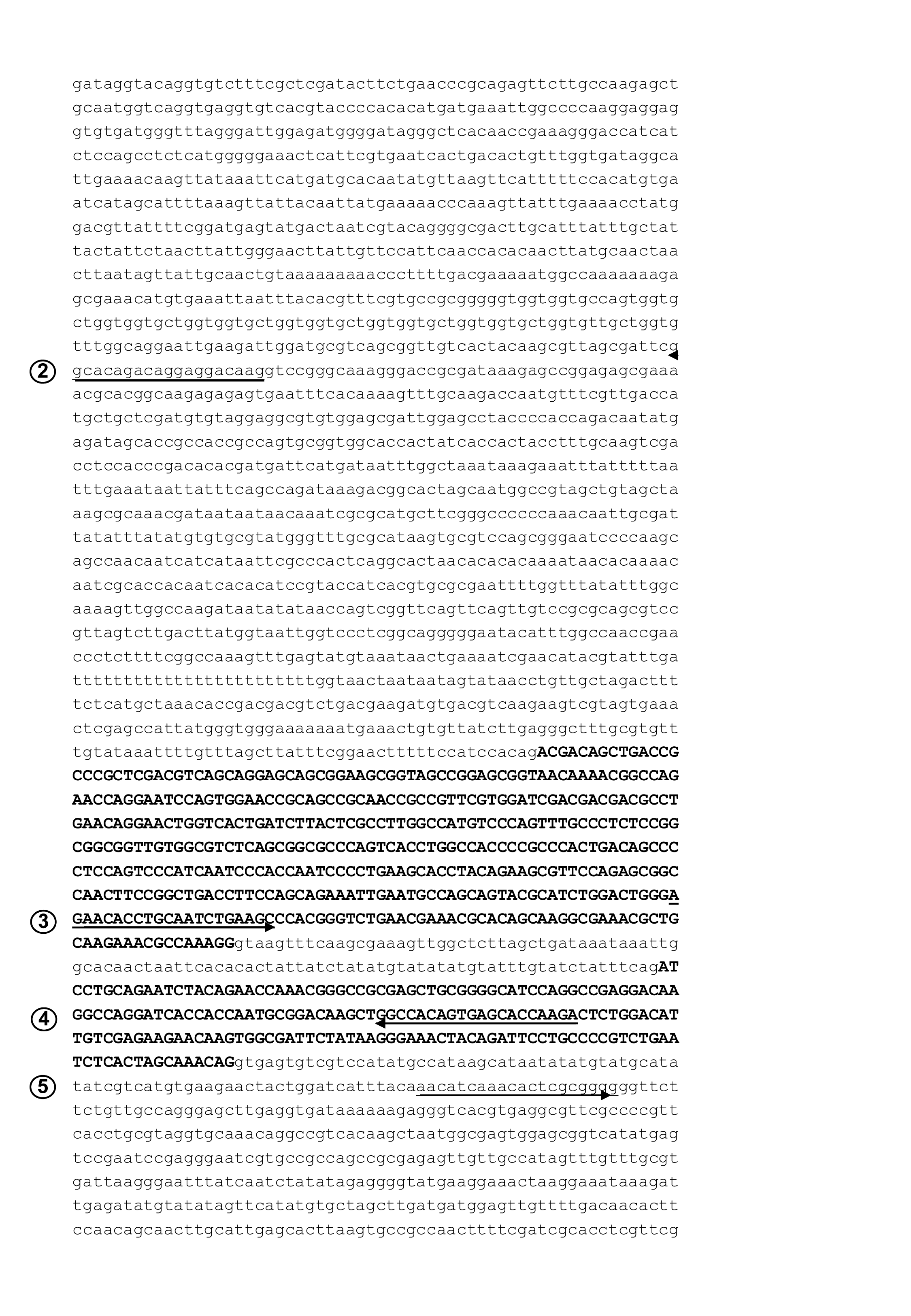 | | --- | |

| | 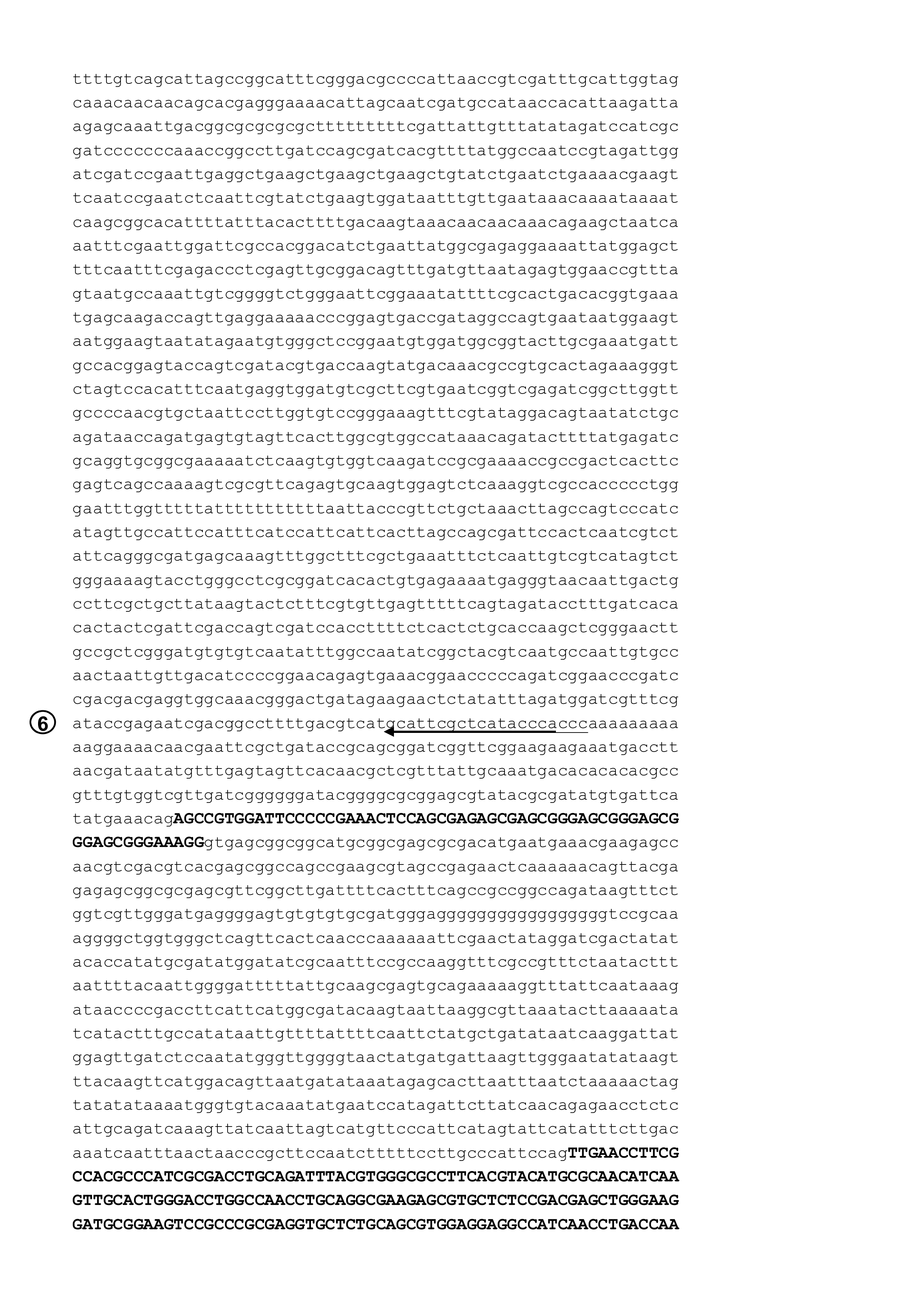 | | --- | | |
| --- | --- | --- |
| | 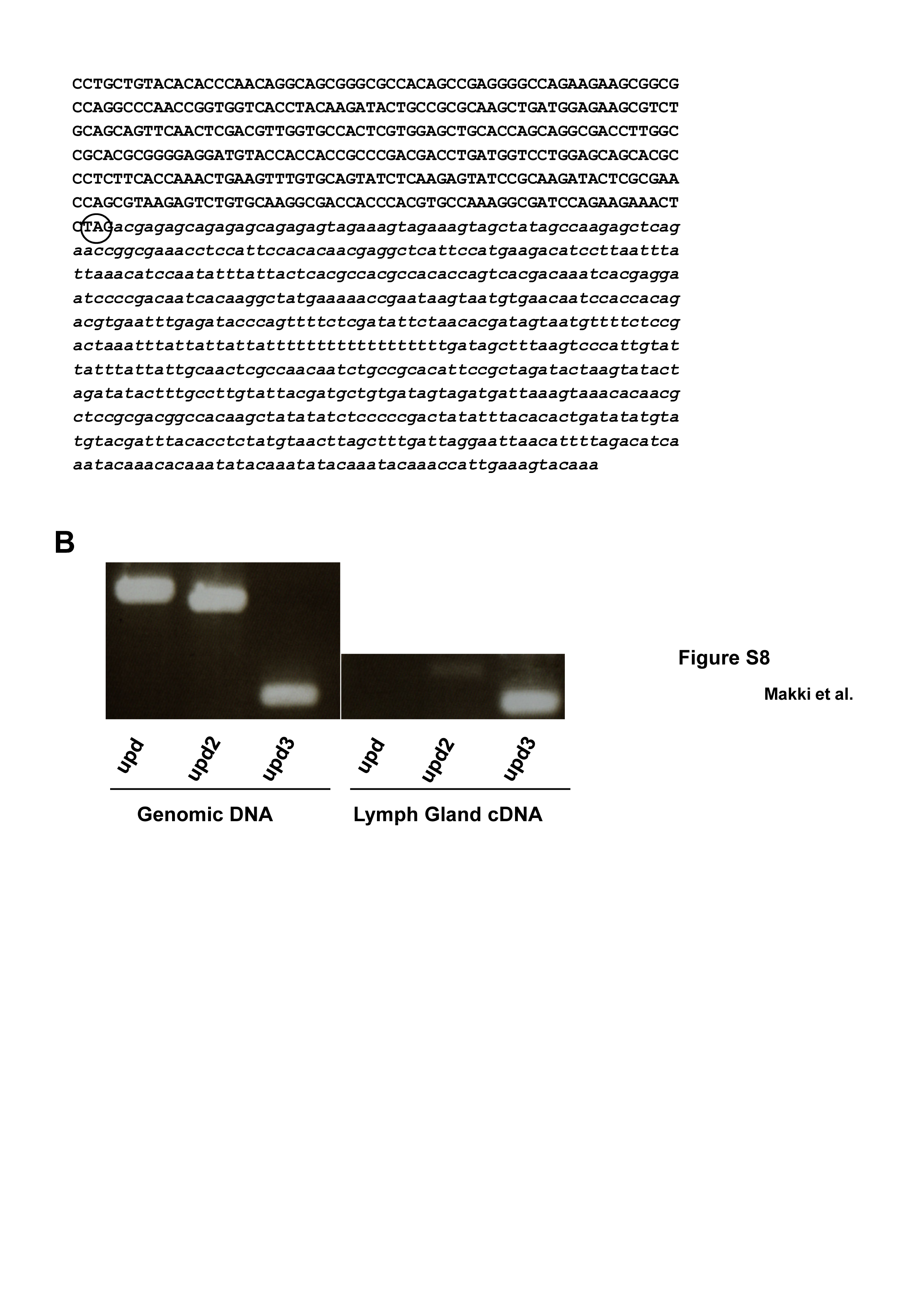 | | --- | |  |

Supplement: Figure S8 — Mapping the upd3 transcription start and initiation codon. (A) Nucleotide sequence of the D. melanogaster upd3 genomic region as extracted from Flybase. ORFs are in bold capital letters, untranslated 5′ and 3′ sequences in bold italic lower case, introns and intergenic regions in lower case. Since only genome annotation data were available for upd3, we verified the 5′ end of upd3 by RACE-PCR, starting from total RNA isolated from larval LGs. The transcription start is indicated by an arrowhead with +1, the translation initiation codon (ATG) underlined, and the stop codon circled. Primers used are underlined and numbered. (B) RT-PCR analysis of upd, upd2, and upd3 expression in LGs. Left, PCR amplification on control genomic DNA; right, PCR amplification from RNA of dissected LG. Only upd3 expression is detected at significant levels. (2.16 MB DOC) [file pbio.1000441.s008.doc]

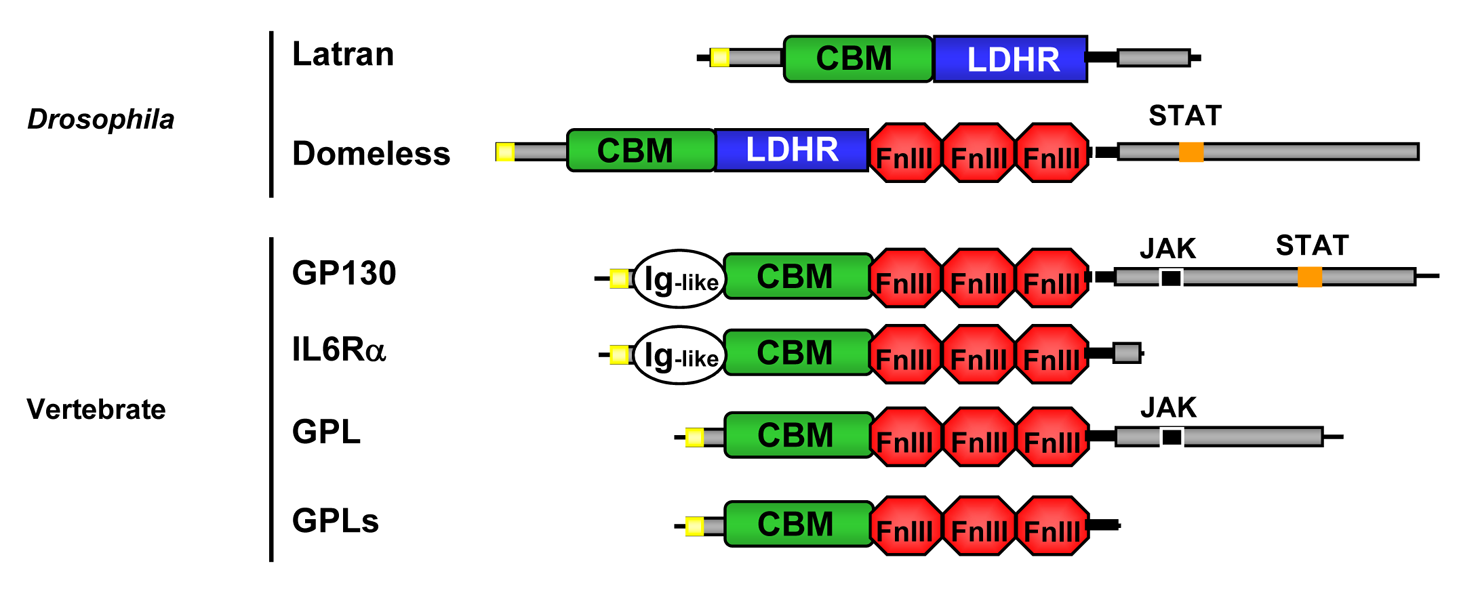

Supplement: Figure S9 — Schematic of type I cytokine receptors from Drosophila and Vertebrates. The green box corresponds to the CBM and the blue box to the LDHR; Fibronectin III (FnIII) motifs are indicated in red; the signal peptide in yellow. TM indicates the transmembrane domain. The intracytoplasmic regions are in grey, with the position of the STAT and JAK binding sites in orange and black, respectively. Ig-like domains are highlighted by circles. (0.21 MB TIF) [file pbio.1000441.s009.tif]
